# The legumain McPAL1 from *Momordica cochinchinensis* is a highly stable Asx-specific splicing enzyme

**DOI:** 10.1016/j.jbc.2021.101325

**Published:** 2021-10-26

**Authors:** Heng Tai Liew, Janet To, Xiaohong Zhang, Xinya Hemu, Ning-Yu Chan, Aida Serra, Siu Kwan Sze, Chuan-fa Liu, James P. Tam

**Affiliations:** 1 School of Biological Sciences, Nanyang Technological University, Singapore, Singapore; 2 IMDEA Food Research Institute, +Pec Proteomics, Campus of International Excellence UAM+CSIC, Old Cantoblanco Hospital, Cantoblanco, Madrid, Spain; 3 Proteored – Instituto de Salud Carlos III (ISCIII), Campus UAM, Cantoblanco, Madrid, Spain

**Keywords:** peptide asparaginyl ligase, asparaginyl endopeptidase, pH-dependent trimodal, asparaginyl ligase, cyclic trypsin inhibitor, McPAL1, AEP, asparaginyl endopeptidase, MCoTI-I/II, *Momordica cochinchinensis* trypsin inhibitor I/II, PAL, peptide asparaginyl ligase, SFTI, sunflower trypsin inhibitor, VPE, vacuolar processing enzyme

## Abstract

Legumains, also known as asparaginyl endopeptidases (AEPs), cleave peptide bonds after Asn/Asp (Asx) residues. In plants, certain legumains also have ligase activity that catalyzes biosynthesis of Asx-containing cyclic peptides. An example is the biosynthesis of MCoTI-I/II, a squash family-derived cyclic trypsin inhibitor, which involves splicing to remove the N-terminal prodomain and then N-to-C-terminal cyclization of the mature domain. To identify plant legumains responsible for the maturation of these cyclic peptides, we have isolated and characterized a legumain involved in splicing, McPAL1, from *Momordica cochinchinensis* (Cucurbitaceae) seeds. Functional studies show that recombinantly expressed McPAL1 displays a pH-dependent, trimodal enzymatic profile. At pH 4 to 6, McPAL1 selectively catalyzed Asp-ligation and Asn-cleavage, but at pH 6.5 to 8, Asn-ligation predominated. With peptide substrates containing N-terminal Asn and C-terminal Asp, such as is found in precursors of MCoTI-I/II, McPAL1 mediates proteolysis at the Asn site and then ligation at the Asp site at pH 5 to 6. Also, McPAL1 is an unusually stable legumain that is tolerant of heat and high pH. Together, our results support that McPAL1 is a splicing legumain at acidic pH that can mediate biosynthesis of MCoTI-I/II. We purport that the high thermal and pH stability of McPAL1 could have applications for protein engineering.

Legumain belongs to the C13 subfamily of cysteine proteases represented by asparaginyl endopeptidase (AEP) and was first found in legume seeds (1, 2). These proteases specifically cleave proteins at Asn/Asp (Asx) sequences. Plant legumains are activated in acidic vacuoles and thus are also referred to as vacuolar processing enzymes (VPEs) (3). Here we focus on the role of legumains in the biosynthesis of plant-derived cyclic peptides containing both Asn- and Asp-processing sites and thus reference these enzymes as legumains.

Legumains are found in both animals and plants (4). In parasite *Schistosoma mansoni*, legumains are involved in the sequential degradation of hemoglobin into diffusible peptides and free amino acids (5). In animals, legumains mainly function in the immune response by processing self and foreign antigens for presentation on the major histocompatibility complex II (MHC-II) complex (4). In plants, legumains are essential for processing seed storage proteins involved in seed maturation and programmed cell death (3, 6, 7). Mammals have only one legumain isoform, whereas plants encode multiple functional isoforms (4). These plant isoforms can be grouped into seed type and vegetative type that reflect their isoform-specific localizations and functions (7, 8).

Legumains are unusual enzymes in that they exhibit three distinct catalytic activities: hydrolase, ligase, and splicing. The hydrolase activity of legumains is mainly involved in activation of seed storage proteins, proteolytic cascades in programmed cell death, and autocatalytic activation. These hydrolytic processes occur in lysosomes or lytic vacuoles, which are both acidic. In addition to the Asn-hydrolytic activity of AEPs (9, 10), AEPs were also known in 1990s as splicing enzymes in the posttranslational modification of concanavalin by mediating circular permutation (11).

Legumain-mediated protein splicing, recognized as early as 1985 to play a role in concanavalin A (ConA) maturation, combines both hydrolytic and ligase activity in tandem during protein processing and involves excision of intervening sequences and religation of the remaining sequences to induce a different circular-permutated structure (12). This posttranslational splicing process mediated by enzyme jack bean AEP was described in 1994 by Min and Jones (11). The ConA precursor contains multiple Asn-cleavage sites for jack bean AEP (CeAEP1) processing. After multiple cleavages at Asn residues, the folded pro-ConA is processed into an N- and C-chain. Proximity-driven ligation then occurs between the N-chain N-terminus and the C-terminal Asn of the C-chain to swap the 1-dimensional order of the two fragments to produce a circular permutation of ConA (Fig. S1) (11).

Recently, an increasing number of legumains have been found to participate in cyclic peptide biosynthesis in two different modes. Firstly, they act as Asx-specific ligases that convert linear peptide precursors into cyclotides that have diverse biological activities. Examples include butelase-1 discovered in *Clitoria ternatea* (13), OaAEP1b from *Oldenlandia affinis* (14), HeAEP3 from *Helianthus enneaspermus* (15), and VyPAL2 from *Viola yedoensis* (16). To date, butelase-1 is the most efficient ligase and can orchestrate site-specific intra- and intermolecular ligation of a diverse range of peptides and proteins under physiological conditions (17). Both butelase-1 and butelase-1-like ligases exhibit Asn-ligase activity at acidic and basic pH (16, 18). These ligases only exhibit a low level of Asn-specific hydrolytic activity at pH 4.5 or lower for autoactivation from their proenzymes. As such, they are collectively referred to as pH-independent peptide asparaginyl ligases (PALs). Secondly, AEPs mediate maturation of cyclic peptide by a splicing mechanism, which is also known as “cleavage-dependent ligation” (19). Examples of these cyclic peptides include sunflower trypsin inhibitor 1 (SFTI-1), orbitides, and the squash family of trypsin inhibitors MCoTI-I and MCoTI-II (MCoTI-I/II) (20).

*Momordica cochinchinensis* belongs to the Cucurbitaceae family and is used as a traditional Chinese medicine. *M. cochinchinensis* produce the cyclic trypsin inhibitors MCoTI-I and MCoTI-II (MCoTI-I/II) that have Asp at the C-terminal processing site and are thought to be processed from a linear precursor by an Asp-specific legumain-ligase (21, 22, 23). Previous studies demonstrated that MCoTI-I/II precursors harbor an N-terminal Asn (DIN↓GG) and a C-terminal Asp (GSD↓AL) processing site (24). A recent *in vitro* study demonstrated that a recombinantly produced AEP from *M. cochinchinensis*, MCoAEP2, processed both the N- and C- terminal domain of the MCoTI-II precursor at acidic pH to produce cyclic MCoTI-II (25).

Here we report the isolation and characterization of an unusual Asx-specific ligase designated McPAL1 from *M. cochinchinensis* seeds. Using a panel of Asx-containing peptides including the MCoTI-II linear precursor and other bioactive peptides, we examined the unusual trimodal catalytic mechanism of McPAL1 that involves pH- and P1-residue-dependent activities that confer its multifunctionality as a splicing enzyme, hydrolase, and ligase. We also characterized the exceptionally high tolerance of McPAL1 to heat and basic pH.

## Results

### Transcriptome analysis of *M. cochinchinensis* legumains

To identify AEPs in *M. cochinchinensis* seeds, seed extracts were first screened for ligase activity using an Asn-containing peptide substrate, GN^12^-GL (Fig. S2). Seed powder from frozen fresh *M. cochinchinensis* seeds was suspended at pH 6 with a cocktail of EDTA, β-mercaptoethanol and serine protease inhibitor. After centrifugation, ligase activity in the clarified crude solution was assessed using GN^12^-GL at room temperature. Incubation with the crude *M. cochinchinensis* seed extracts yielded the desired cyclic cGN^12^ and linear peptide GN^12^, thus confirming the presence of legumain(s) in *M. cochinchinensis* seeds (Fig. S2).

To determine the corresponding legumain sequences, we obtained a transcriptome assembly of *M. cochinchinensis* using total RNA isolated from fresh seeds that was sequenced by the Beijing Genomics Institute (BGI). The resulting transcriptome was deposited under NCBI SRA accession no. PRJNA655570. Precursor sequences of butelase-1 and *Oa*AEP1b were used to search for sequences homologous to those of legumains. Four legumain precursors, designated McPAL1-4, were found in the *M. cochinchinensis* transcriptome.

Pairwise global alignment showed that McPAL1 and McPAL2 share 54.2% overall sequence identity and 67.1% core-domain identity (Fig. S3). Compared with butelase-1, McPAL1 and McPAL2 respectively have 50.5% and 66.0% overall sequence identity, and 59.9% and 67.1% core-domain sequence identity. McPAL3 had a lower degree of sequence identity to McPAL1-2. Alignment of the full-length sequences for McPAL1-4 with that of *C. ternatea* butelase-1 and butelase-2, as well as human legumain (LGMN), revealed that McPAL1-4 carry the catalytic triad of the legumain C13 family comprising Asn70, His175, and Cys217 (numbering according to McPAL1). In addition, McPAL1-3 exhibit signature zymogen legumain domains consisting of a signal peptide, N-terminal domain, AEP core domain, linker domain, and cap domain. McPAL4 is a truncated sequence. Together, these results confirmed that McPAL1-3 are new members of the legumain family.

Du *et al*. identified two AEPs, MCoAEP1 and MCoAEP2, from *M. cochinchinensis* transcriptomes (25). We compared McPAL1 and McPAL2 with MCoAEP1 (accession no. MK770254) and MCoAEP2 (accession no. MK770255) sequences using pairwise sequence alignment (Fig. S4), which showed that McPAL1 has 99.2% identity with MCoAEP1, which was not characterized by Du *et al.* due to its low expression levels in a bacterial system. McPAL1 differs from MCoAEP1 at two core domain residues and two cap domain residues. McPAL2 and MCoAEP2 differed by ten residues: five in the core domain, one in the linker region, and three in the cap domain. Given that most of these variations are outside the active site and substrate-binding pockets of these ligases, they likely have minimal impact on enzymatic activity.

### Isolation, identification, and characterization of native McPAL1 from *M. cochinchinensis* seeds

To isolate the first native legumain from the Cucurbitaceae family, fresh *M. cochinchinensis* seeds were homogenized and subjected to a two-step purification procedure including ion-exchange chromatography and size-exclusion chromatography. The purified enzyme had an apparent molecular weight of 30 kDa as determined by SDS-PAGE (Fig. S5).

To determine the composition of the isolated ligases, we performed in-gel trypsin and chymotrypsin digestion followed by LC-MS/MS sequencing. The transcriptome of *M. cochinchinensis* seed extract was used for database search using PEAKS studio (Version 7.5). McPAL1 proenzyme sequence has been identified with close to 75% core-domain coverage (Table S1, Fig. S6). Only two peptides were from McPAL2 and no fragments matched any region of McPAL3 or McPAL4. In addition, we determined a seed lectin coeluted with McPAL1 by MALDI-TOF-TOF MS/MS analysis (Fig. S7). Thus, we concluded that McPAL1 is likely the most abundant legumain in *M. cochinchinensis* seeds.

### Production of recombinant McPAL1 from *E. coli* and insect cells

**Figure 1:**
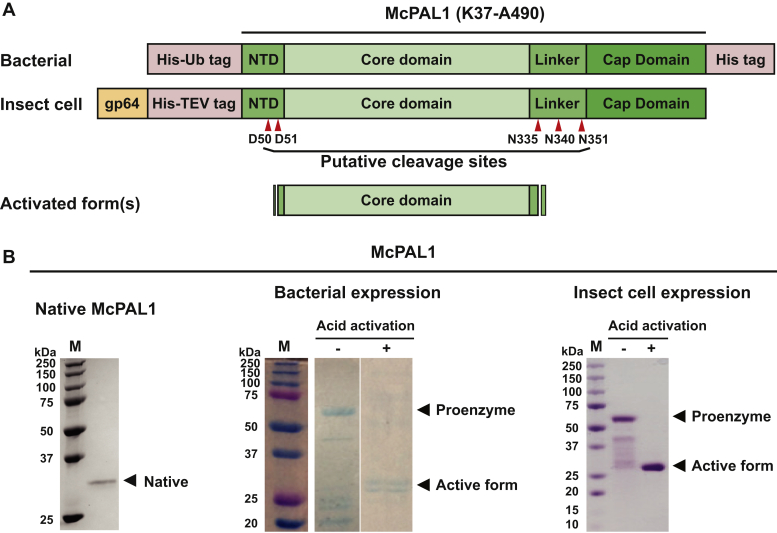
**Recombinant expression and activation of McPAL1.***A*, schematic representation of the bacterial and insect cell constructs used in this study, with the predicted protein domains indicated. The insect cell constructs included an additional N-terminal gp64 secretion signal to facilitate McPAL1 secretion. *B*, gel profiles of native McPAL1 isolated from *M. cochinchinensis* seeds and recombinant McPAL1 obtained from bacterial and insect cell expression systems, with McPAL1 proenzymes and mature enzymes indicated. Appearance of two active form bands in the bacterial-expressed McPAL1 may be due to processing at different Asn/Asp cleavage sites during autoactivation, while for the insect cell-expressed McPAL1, the additional site(s) may be protected by glycosylation. The purity of protein fractions eluted from metal affinity, anion exchange, and size-exclusion chromatography was analyzed by SDS-PAGE and Coomassie Blue staining.

To characterize McPAL1, we expressed an unglycosylated and glycosylated form in bacteria and insect cell systems, respectively (Experimental procedures). The gene encoding the complete McPAL1 amino acid sequence was cloned into the respective expression vectors, with a His-tag for affinity purification substituted for the signal peptide (Fig. 1*A*). Following a three-step chromatographic purification process that involved metal-affinity, anion exchange, and size-exclusion chromatography (Experimental procedures), the bacterial and insect cell systems yielded 1.5 and 5 to 10 mg/l, respectively, of purified proenzymes.

We converted the McPAL1 proenzyme into the mature activated form using acid-activation that involves incubating the McPAL1 proenzyme at pH 4.5 for 16 h at 4 °C in the presence of 0.5 mM N-lauroylsarcosine, 5 mM β-mercaptoethanol, and 1 mM EDTA. This technique was also previously used for human and plant legumains for which an acidic pH environment promotes autoactivation of the proenzyme into its fully activated form (26, 27). Activated McPAL1 was further purified by size-exclusion chromatography (Fig. 1*B*). Good results were achieved for McPAL1 expressed from *Sf9* insect cells, and recombinant McPAL1 produced from *Sf9* cells was used for subsequent experiments.

### Substrate design to determine McPAL1 specificity and splicing mechanism

MCoTI-I/II biosynthesis requires a splicing mechanism involving first an Asn-specific endopeptidase activity and then Asp-preferring ligase activity. This cleavage-coupled ligation requires an N-terminal cleavage site in the MCoTI-I/II precursors with an Asn and Asp at the P1 positions of the N-terminal tripeptide (DIN) and C-terminal ligation site (DAL), respectively.

To gain insight into the potential splicing activity of McPAL1, we prepared a panel of P1-Asx peptides having different lengths and sequences that mimic the linear precursor of MCoTI-II (Table 1). The Asp-containing model peptide substrate GLRRGYSGSDAL (GD^10^-AL) contains the C-terminal hexapeptide SGSDAL of the MCoTI-II precursor. The tripeptide DAL is conserved in MCoTI peptides and serves as the recognition motif for legumains (24). For comparison, we also prepared a similar shortened version of MCoTI-II having an Asn at P1, GLRRGYSGSNAL (GN^10^-AL), which contains the NAL-tripeptide motif.

**Table 1:** McPAL1-mediated ligation on Asx-containing peptide substrates

Peptide substrate			Reaction conditions		Products^a^	
Name	Sequence	Ring size^b^ (a.a.)	pH	Time (h)	Cyclic	Linear
GD^10^-AL	GLRRGYSGSDAL^c^	10	5^d^	0.7	61 ± 4	7 ± 1
GN^10^-AL	GLRRGYSGSNAL^c^	10	5^d^	0.5	55 ± 15	42 ± 18
DIN-GD^10^-ALEG	DINGLRRGYSGSDALEG	10	5^d^	1.0	58 ± 5	41 ± 5^e^
DIN-MCoTI-II-ALEG	DINGGVCPKILQRCRRDSDCPGACICRGNGYCGSGSDALEG	34	5	3.0	95 ± 2	N.D.^f^
GN^10^-AL	GLRRGYSGSNAL^c^	10	7^d^	0.5	93 ± 4	N.D.
KN^14^-GL-pB1	KLGTSPGRLRYAGNGL	14	7	0.2	91 ± 1	N.D.
MrlA	GVCCGYKLCHPCAGNGL	15	7	0.2	90 ± 3	5 ± 2
Hylaseptin	GILDAIKAIAKAAGNGL	15	7	0.2	77^g^	9
RN^6^-HV	RLYRGNHV	12	7	2.0	55 ± 2 (11 ± 1)^h^	N.D.

**Figure 2:**
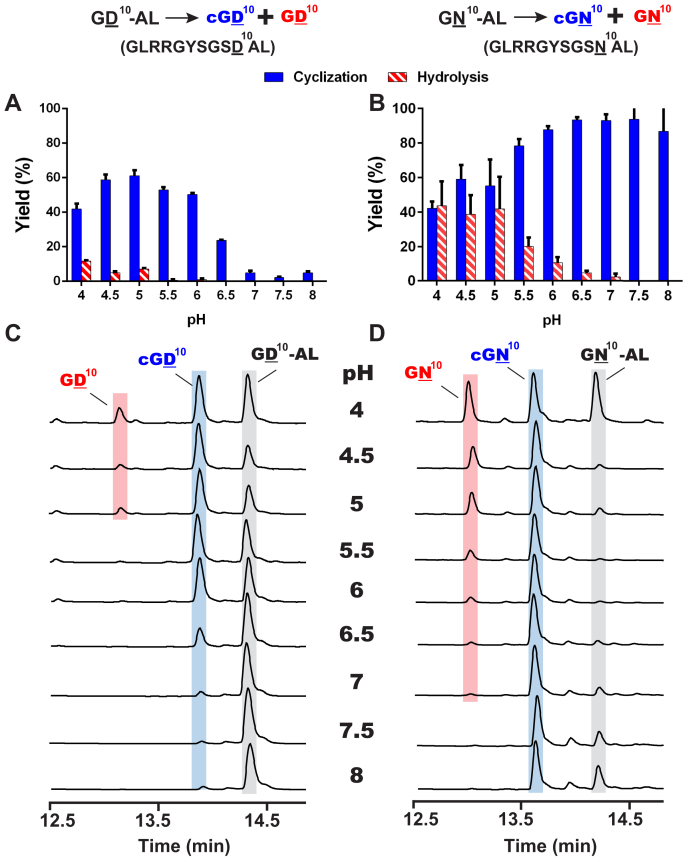
**Product analysis of P1-Asp- and P1-Asn-containing GD**^**10**^**-AL and GN**^**10**^**-AL peptides treated with McPAL1 expressed from *Sf9* insect cells for 30 min.** Quantitative product analysis of McPAL1-mediated reactions of (*A*) GD^10^-AL and (*B*) GN^10^-AL in the pH range of 4 to 8. *C* and *D*, HPLC profiles of McPAL1-mediated reactions of GD^10^-AL and GN^10^-AL. All reactions were performed using 1:500 McPAL1: substrate in 0.5 mM TCEP (or DTT) in sodium citrate (or sodium phosphate) buffer at 37 °C for 12 to 30 min (1:100 McPAL1: substrate for GD^10^-AL and 1:500 for GN^10^-AL). Average yield and standard deviations (SDs) were calculated from experiments performed in triplicates.

**Figure 3:**
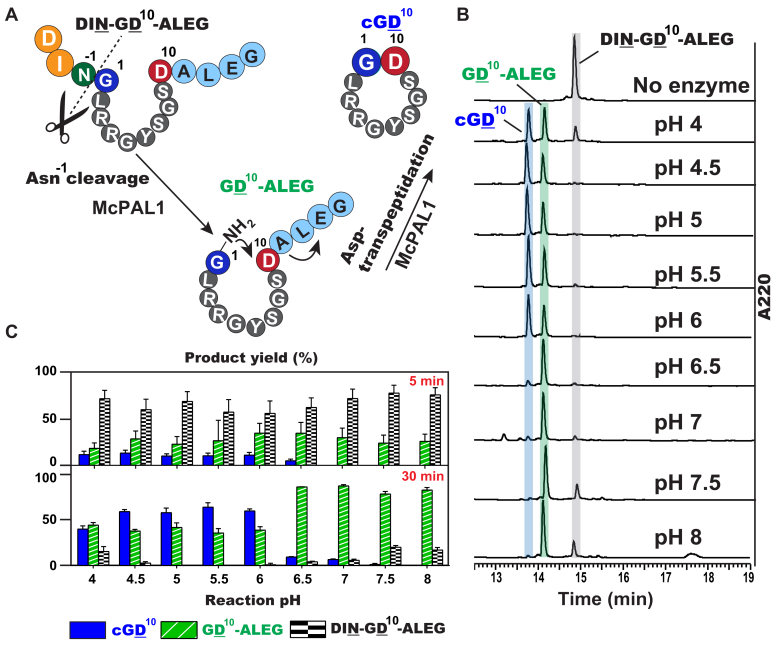
**HPLC analysis of *Sf9*-expressed McPAL1-mediated processing of the model peptide DIN-GD**^**10**^**-ALEG.***A*, schematic representation of McPAL1-mediated Asn^−1^ cleavage and Asp^10^ cyclization. *B*, HPLC profiles of McPAL1-mediated processing of DIN-GD^10^-ALEG from pH 4 to 8. *C*, quantitative product analysis of McPAL1-mediated processing of DIN-GD^10^-ALEG from pH 4 to 8 for 5 min and 30 min. Cyclic cGD^10^ and linear GD^10^-ALEG are shaded in *blue* and *green*, respectively. The starting material DIN-GD^10^-ALEG is shaded in *gray*. Average yield and standard deviations (SDs) were calculated from experiments performed in triplicates.

P1 Asx residues are underlined.

a Average yield and standard deviations (SDs) were calculated based on experiments performed in triplicates.

b Number of amino acids in the cyclic products.

c Model peptide sequence derived from MCoTI-I/II (--GYSGSDAL).

d pH profile from pH 4 to 8, see Figures 2 and 3.

e GD^10^-ALEG (41%).

f N.D. Not detected in HPLC profiles.

g Based on single experiment.

h Cyclodimer (55 ± 2%) and cyclotrimer (11 ± 1%).

To examine McPAL1 processing and cyclizing activity toward the MCoTI-II precursor, we synthesized a mini-MCoTI-II bi-Asx-substrate (DIN-GD^10^-ALEG) and a full precursor of MCoTI-II (DIN-MCoTI-II-ALEG) containing both Asn and Asp residues at the N- and C-terminal processing sites, respectively. The mini-MCoTI-II peptide precursor was designed as a flexible peptide precursor that lacks the three cross-strand disulfides that provide conformational constraint to the MCoTI-II substrate.

Peptides having sequences unrelated to that of MCoTI-II were also synthesized to show the scope of substrates in McPAL1-catalyzed reactions (Table 1). Since macrocycles having between 10 and 20 amino acids have a high potential for peptidyl drug development, most model peptides used here were in this size range and could form cyclic peptides (28).

### pH-dependent trimodal profile of McPAL1

Next, we determined the enzymatic profile of McPAL1 between pH 4 and pH 8 using GD^10^-AL and GN^10^-AL, a pair of substrates that differ only in their P1 sites (D *versus* N) (Table 1, Fig. 2). The reaction products were analyzed by C-18 reverse-phase HPLC (RP-HPLC) and MALDI-TOF.

In the acidic range between pH 4 and 6.5, McPAL1 acted mainly as an Asp-ligase on the Asp-containing GD^10^-AL peptide to yield the cyclic cGD^10^ (Fig. 2, *A* and *C*). In contrast, McPAL1 acted bidirectionally on the Asn-containing substrate GN^10^-AL to yield both the hydrolyzed linear product GN^10^ and cyclic product cGN^10^ (Fig. 2, *B* and *D*). At pH 5, 42% of linear GN^10^-AL and 7% of linear GD^10^-AL were detected, and 57% of cGN^10^ and 61% of cGD^10^ were detected (Table 1). Although P1-Asp can shift the equilibrium toward ligation (29), the predominant Asp-ligase activity of McPAL1 is uncommon for plant legumains. For example, AtLEGγ is a stronger Asp-hydrolase than Asp-ligase at acidic pH (10).

The reaction profile changed when the pH was adjusted from neutral to basic pH (*i.e.*, pH 7–8). At pH 7, McPAL1 acted predominantly and efficiently as an Asn-ligase to yield ≥90% cyclic cGN^10^ from GN^10^-AL. The catalytic efficiency (K_cat_/K_m_) of McPAL1-mediated cyclization of GN^10^-AL at pH 7 was estimated to be 1.3 × 10^5^ M^−1^s^−1^ (Fig. S8). Within the same pH range, McPAL1 continues to act as an Asn-ligase, but was largely inactive toward GD^10^-AL, with >90% of the starting peptide remaining after 1 h. The turnover rate (k_cat_) of the GD^10^-AL cyclization reaction was 0.98 s^−1^ at pH 5, and this was nearly 50-fold slower than that of GN^10^-AL cyclization at pH 7 (48 s^−1^) (Fig. S8). This result suggests that protonation of an incoming amine nucleophile (−NH_3_^+^) at acidic pH reduces the ligation efficiency of McPAL1 compared with the deprotonated incoming amine (−NH_2_) present at basic pH.

Comparison of McPAL1 expressed from *Escherichia coli* and insect cells showed that both forms had similar pH-dependent profiles. With the P1-Asn substrate GN^12^-GL, linear GN^12^ was the major product of McPAL1 produced from *E. coli* at acidic pH, while the cyclic product cGN^12^ predominated at basic pH (Fig. S9*A*). Similarly, for *Sf9*-expressed McPAL1, 37% and <5% of GN^12^ were observed at pH 5 and pH 7, respectively (Fig. S9*B*).

Together, our results indicated a trimodal profile of McPAL1 activity. At acidic pH, McPAL1 shows a bimodal profile by acting as an Asn-specific hydrolase and an Asp-specific ligase. At basic pH, McPAL1 is monomodal, acting predominantly as an Asn-ligase for Asn-containing peptides.

### Processing of MCoTI-II precursors by McPAL1-mediated splicing at acidic pH

To show that the bimodal catalytic action of McPAL1 at acidic pH is shifted toward splicing activity, we used the mini-MCoTI-II bi-Asx-substrate DIN-GD^10^-ALEG (Fig. 3*A*). We expected that under acidic pH conditions, McPAL1 would first cleave the substrate at the N-terminal tripeptide (DIN-) and then cyclize at the C-terminal P1-Asp site. Indeed, we observed McPAL1-mediated splicing of DIN-GD^10^-ALEG to form the expected cyclic product cGD^10^ with both ends trimmed within a narrow range of pH 4 to 6 (Fig. 3, *B* and *C*). The optimal condition for McPAL1-mediated splicing of DIN-GD^10^-ALEG was pH 5.5, for which a 60% yield of cGD10 within 30 min was obtained. At pH 6.5 and above, the cGD^10^ yield decreased significantly to <10%, and accumulation of intermediate GD^10^-ALEG was observed.

**Figure 4:**
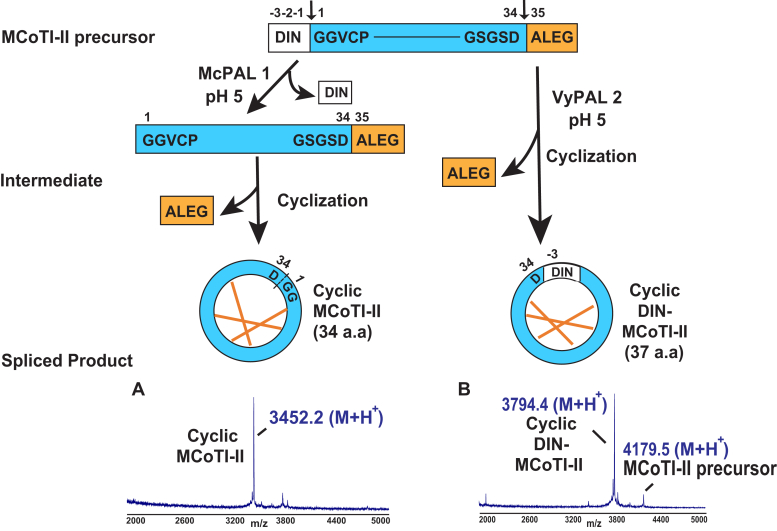
**Schematic representation of N- and C-terminal splicing of MCoTI-II precursor by McPAL1 and VyPAL2 at pH 5.** McPAL1 cleaved the MCoTI-II precursor at both termini and resulted in MCoTI-II cyclization after 12 h at pH 5 at 30 °C. *A*, the amino acid residues Asn^−1^ and Asp^34^ are the cleavage sites for MCoTI-II precursor maturation mediated by McPAL1, resulting in cyclization and production of cyclic MCoTI-II containing 34 amino acids. *B*, reaction of VyPAL2 with MCoTI-II precursor generated cyclic DIN-MCoTI-II containing 37 amino acids. The reaction products were analyzed by MALDI-TOF mass spectrometry.

Next, we used McPAL1 to mediate splicing of the native MCoTI-II precursor DIN-MCoTI-II-DALEG, which contains the folded and disulfide-constrained MCoTI-II domain flanked by DIN and ALEG at the N- and C-termini, respectively (Fig. 4). At pH 5, McPAL1 first cleaved after Asn^−1^ of the N-terminal tripeptide DIN to produce the desired intermediate MCoTI-II-DALEG and then cyclizes the intermediate at the C-terminal P1-Asp of cyclic MCoTI-II (Fig. 4).

We also compared the pH-dependent ligase activity of McPAL1 with that of two pH-independent ligases, VyPAL2 and butelase-1. These two PALs act as efficient cyclases against GN^10^-AL and GD^10^-AL and have activity that is largely unaffected at pH 5 to 8 (Fig. S10, *A* and *B*).

For processing of the MCoTI-II precursor DIN-MCoTI-II-DALEG, VyPAL2 acted monomodally to produce cyclic DIN-MCoTI-II at pH 5, and little Asn-hydrolase activity to remove the N-terminal tripeptide DIN was observed (Fig. 4). On the other hand, no butelase-1-mediated processing of the MCoTI-II precursor was seen at pH 5 even after 12 h (Fig. S10*C*). Taken together, McPAL1 acts as a splicing enzyme at pH 5, and in this regard, its splicing activity is unique from PALs such as VyPAL2 and butelase-1.

### Cyclization of bioactive peptides by McPAL1-mediated ligation at neutral to basic pH

McPAL1 is an efficient Asn-specific ligase at pH 7 for a panel of peptides of various lengths (Table 1). Peptides in this panel included KN^14^-GL-pB1, MrIA, and hylaseptin, which are derived from the bleogen pB1 of *Pereskia bleo* from the Cactaceae family (30), conotoxin (MrIA) from marine cone snail, and hylaseptin-P1 from *Hyla punctate* (31), respectively (Fig. S11). McPAL1 efficiently catalyzed N-to-C cyclization of these peptides with >75% yield achieved within 12 min.

In addition, McPAL1 efficiently produced a cyclodimer of the RLYR-containing peptide precursor RN^6^-HV derived from protegrin PG-1 and tachylepsin TP-1, which both have known antimicrobial activity (Table 1, Fig. S12) (32, 33). RN^6^-HV was synthesized with a His-Val leaving group that is favored by butelase-1. At 1 mM RN^6^-HV, a 65% yield of cyclodimer and 13% cyclotrimer were obtained following McPAL1 treatment for 2 h at pH 7. The results show that McPAL1 retained high reactivity toward other dipeptide leaving groups favored by PAL, particularly at pH >6.5 and with Asn at P1.

### McPAL1 exhibits high stability against heat and basic pH

**Figure 5:**
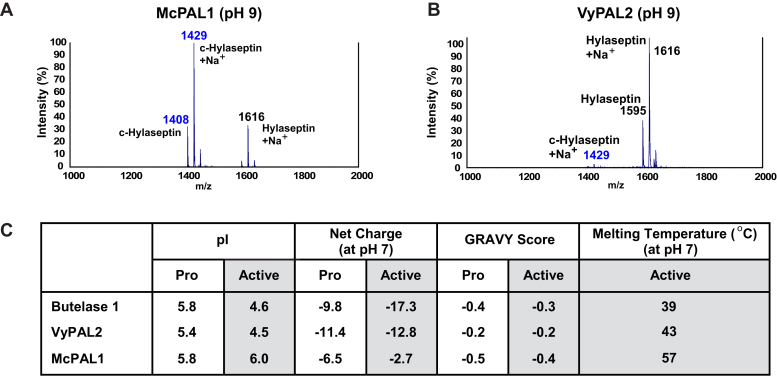
**Thermal stability, isoelectric point (pI), and net charge of McPAL1in comparison with butelase-1 and VyPAL2.***A* and *B*, cyclization of hylaseptin by McPAL1 and VyPAL2 at pH 9. The results were analyzed by MALDI-TOF mass spectrometry. *C*, pI, net charge (at pH 7), GRAVY score, and average melting temperature 57 ± 1 °C (at pH 7, n = 4) for McPAL1.

Legumains are generally known to be unstable at neutral to basic pH (26, 34). Most PALs are inactivated at pH >8. In our screening assays conducted during the discovery phase for McPAL1, we found that McPAL1 was exceptionally tolerant to very high pH; it retained high activity to cyclize hylaseptin at pH 9, compared with VyPAL2, which is almost fully inactivated at the same pH (Fig. 5, *A* and *B*). Based on this observation, we then explored the pH-dependent thermal stability of McPAL1. Active McPAL1 showed pH-dependent thermal stability, with the highest T_m_ recorded in the pH range from 4.5 to 6 (Fig. S13*A*). At neutral to basic pH, McPAL1 is more stable compared with other PALs reported to date (Fig. 5*C*). The melting temperature (T_m_) of McPAL1 at pH 7.0 is 57 ± 1 °C, which is ≥15 °C higher than other PALs such as butelase-1 and VyPAL2. We also analyzed biophysical properties of McPAL1 including isoelectric point (pI), net charge, and hydrophobicity (GRAVY score) in comparison with the other two PALs (Figs. 5*C* and S13*B*). The relatively high pI (6.0) of McPAL1 active form compared with butelase-1 (pI = 4.6) and VyPAL2 (pI = 4.5) could be the main contributor to its higher thermal stability under basic pH. This relatively higher pI is attributed to the presence of more positively charged residues and less negatively charged residues in the active form of McPAL1 relative to butelase-1 and VyPAL2 (Fig. S14). Reducing the negative/positive ratio of surface residues has been previously shown to improve the resistance of an enzyme at alkaline pH conditions (35). The similar GRAVY scores for the three enzymes suggested that hydrophobic interactions are not a major differentiating factor for the observed pH-dependent thermal stability of McPAL1.

**Figure 6:**
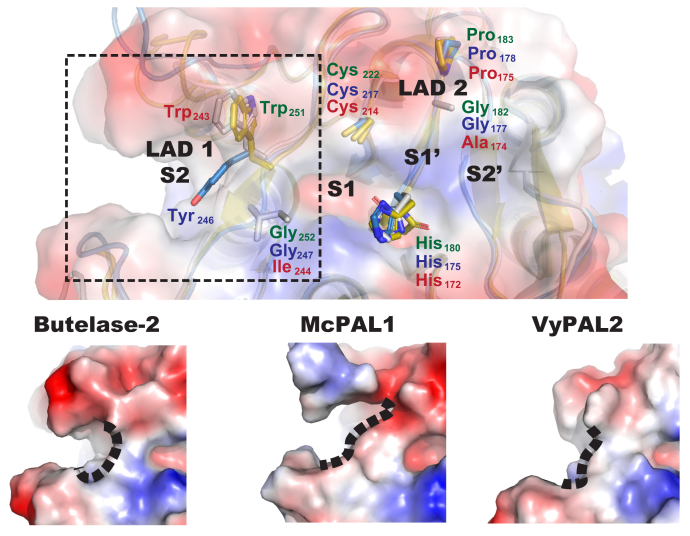
**Molecular models of butelase-2 and VyPAL2 catalytic profiles and the trimodal catalytic profile McPAL1.** Substrate-binding pockets of butelase-2, McPAL1, and VyPAL2. LAD1, LAD2, and the catalytic triads are represented as sticks and respectively colored in *yellow* (butelase-2), *cyan* (McPAL1), and *gray* (VyPAL2). The molecular surface of butelase-2 is represented. The overall conformation of McPAL1 has greater similarity to PALs like VyPAL2.

To investigate the underlying molecular basis of the unusual trimodal enzymatic nature of McPAL1, we compared a model structure of the McPAL1 substrate binding with crystal structures of other legumains and PALs (Fig. 6). In previous studies on VyPAL2 and butelase-2, we introduced and validated the concept of ligase activity determinants (LADs), which are two motifs that flank the S1 site and significantly affect enzymatic directionality (9, 16). LAD1 is a tripeptide motif that shapes the S2 pocket. The first residue of the LAD1 tripeptide is often an aromatic residue. In PALs, the middle residue (also termed “Gate-keeper”) of LAD1 is a hydrophobic residue such as Val, Ile, or Cys, which plays a major role in the ability of PALs to change the substrate acyl-enzyme conformation to one that favors an amine nucleophile rather than water (9). In contrast, most proteolytic legumains have a Gly at this position. In McPAL1, Gly247 occupies the gate-keeper position, but the overall conformation of McPAL1's LAD1 motif, composed of YGT (Fig. 6), is more similar to that of PALs (VyPAL2) compared with proteolytic legumains such as butelase-2. As such, we speculate that the LAD1 motif, which plays a gate-keeper role in McPAL1, may also have synergistic effects on neighboring residues, contributing to its trimodal enzymatic profile. LAD2 is a dipeptide motif near the S1′ pocket. In PALs, LAD2 is AP/GA/AA, but in AEPs is commonly GP. The slightly increased hydrophobicity by the inclusion of one or two Ala residues is believed to favor access of peptides rather than water. McPAL1 adopts a typical proteolytic legumain LAD2 as Gly-Pro, which could explain the Asn-hydrolase activity of McPAL1 at acidic pH.

Notably, the S2′ pocket of McPAL1 is exceptionally hydrophobic. The P2′-S2′ hydrophobic interaction is proposed to be critical for the Asx-ligase activity of legumains by prolonging the retention time of the leaving group and blocking access of water to the S1 catalytic center (29). Therefore, it is reasonable to hypothesize that the strong P2′-S2′ interaction between a substrate and McPAL1 could partially compensate for the hydrophobicity of its LAD2 to further facilitate ligase activity. Taken together, McPAL1 displays enhanced Asx-ligase activity compared with other legumains and maintains Asn-hydrolase activity at acidic pH, which make McPAL1 a unique trimodal enzyme.

## Discussion

This study reports the discovery and characterization of a splicing legumain designated McPAL1 that was isolated from the squash plant *M. cochinchinensis* and is involved in the production of trypsin inhibitors MCoTI-I and MCoTI-II. We have successfully expressed McPAL1 using both the *E. coli* and *Sf9* insect cell systems. While we do not observe any significant difference in the enzymatic activity of McPAL1 from these two expression systems where they produce different glycosylation states, we have mainly used McPAL1 obtained from insect cell expression in this work as it affords higher expression yield than *E. coli*. The surface glycosylation may increase the molecular stability particularly against Asn-specific proteolysis and therefore led to a higher expression yield.

**Figure 7:**
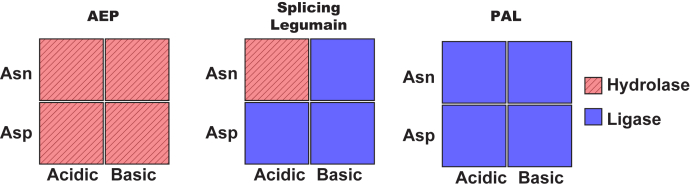
**Schematic diagram summarizing the catalytic profiles of AEPs (*e.g.*, butelase-2), splicing legumains (*e.g.*, McPAL1), and PALs (*e.g.*, butelase-1 and VyPAL2)**.

McPAL1 splicing activity mediates maturation of MCoTI-II from its linear precursor. This splicing activity occurs in a narrow pH range of pH 4 to 6 that favors Asn-hydrolysis and Asp-ligation. At pH >6, McPAL1 Asp-ligation is the rate-limiting step since S1 pocket binding is reduced by deprotonation of the P1-Asp side chain (4). The combination of an N-terminal Asn and a C-terminal Asp at processing sites during MCoTI-I/II splicing also occurs in the maturation of SFTI-1 and PawL1-type orbitides from preproalbumin precursors as reported by Mylne and colleagues (36, 37). These evolutionarily distant examples suggest that a legumain-mediated mechanism of Asn- and Asp-specific tandem processing could be a common strategy for cyclic peptide maturation in acidic vacuoles of plants. To our knowledge, this is the first report of isolation and characterization of a native and dual-functional AEP from the cucumber family that can act as a splicing enzyme. In contrast to canonical legumains that have predominant proteolytic activity at acidic pH (AEP, Fig. 7 left panel), or PALs that have pH-independent ligase activity (PAL, Fig. 7 right panel), McPAL1 can act as a splicing legumain that displays a trimodal catalytic profile (Fig. 7 middle panel). Under acidic conditions, McPAL1 catalyzes hydrolysis of Asn-Xaa bonds and forms Asp-Xaa bonds through Asp-specific ligation. At neutral-to-basic pH, the catalytic profile of McPAL1 reverses to catalyze formation of only Asn-Xaa bonds (Fig. 7). The pH-dependent trimodal activity of McPAL1 suggests that it has different functions in different cellular compartments, including, but not limited to, cleaving, ligating, and splicing of target proteins *in vivo* to enhance the molecular diversity of *M. cochinchinensis* tissues. Furthermore, this particular trimodal activity provides new possibilities for orthogonal ligation with other ligases including PALs (31, 38, 39, 40, 41, 42, 43), as well as chemo-enzymatic peptide and protein labeling (44).

Cyclization and cyclo-oligomerization of nonstructural P1-Asx bioactive peptides *via* McPAL1 suggests that McPAL1 could be a promising tool for peptidyl drug development. Peptide macrocycles having 10 to 20 amino acid residues are gaining interest for drug discovery because of their enhanced metabolic stability against proteolytic degradation and their larger footprint compared with small molecules that together could minimize off-target side effects (45). Furthermore, the unusually high thermal stability and tolerance to basic pH suggest that McPAL1 could have value for protein/peptide bio-orthogonal labeling and precision biomanufacturing at pH >8.

In conclusion, McPAL1 is an unusually stable Asx-ligase that displays a pH-dependent trimodal enzymatic profile. At acidic pH, McPAL1 acts first as a splicing enzyme to form a cyclic peptide by removing both the N(Asn)- and C(Asp)-termini of the precursors flanking the mature domain and then as a ligase to promote head-to-tail cyclization. At basic pH, McPAL1 acts predominantly as an Asn-ligase. Thus, the discovery and characterization of McPAL1 provide insights into the biosynthesis of cyclic peptides and its potential value as a tool for synthesis, semisynthesis, and modification of peptides and proteins.

## Experimental procedures

### Screening of Asx-preferring ligases from *M. cochinchinensis* seeds

One gram of fresh, decoated *M. cochinchinensis* seeds was snap-frozen in liquid nitrogen and then ground into a powder. The powder was extracted with 15 ml extraction buffer (50 mM sodium phosphate buffer, 1 mM EDTA, 5 mM β-mercaptoethanol, 1 mM PMSF, pH 6.0) for 30 min on ice. After centrifugation, the supernatant was collected. The extract supernatant was then mixed 1:10 (v/v) with 25 μM of the peptide substrate GLYRRGRLYRRNGL in 20 mM sodium phosphate buffer containing 5 mM β-mercaptoethanol, pH 6.0 for 5 min at room temperature. The relative ligase activities were analyzed by mass spectrometry based on the formation of the cyclic peptide GLYRRGRLYRRN.

### Isolation of native McPAL1 from *M. cochinchinensis* seeds

Fresh *M. cochinchinensis* seeds (100 g) were homogenized in 500 ml of the abovementioned extraction buffer. Large cellular debris was removed with cheesecloth, and small particles and insoluble materials were removed by centrifugation at 15,000*g*, 4 °C for 20 min. The extraction was conducted at 4 °C to minimize protein degradation. After centrifugation, the supernatant was applied to a flash column containing a 100 ml slurry of SP-Sepharose Fast Flow cation-exchange resin (GE Healthcare). The column was washed with buffer A (50 mM citrate buffer, 1 mM EDTA and 5 mM β-ME, pH 5.0) and eluted with buffer B (50 mM citrate buffer, 1 mM EDTA, 5 mM β-ME and 100 mM NaCl, pH 5.0). The eluent was placed in 10-kDa cutoff dialysis tubing and dialyzed overnight against 12 l of buffer A. The dialyzed sample was filtered and loaded onto four 5 ml HiTrap SP Sepharose high-performance columns connected in series (GE Healthcare) using an ÄKTA FPLC platform with a linear gradient of 0 to 1 M sodium chloride at a flow rate of 5 ml/min (buffer A: 50 mM citrate buffer, 1 mM EDTA and 5 mM β-ME, pH 5.0; buffer B: 50 mM citrate buffer, 1 mM EDTA, 5 mM β-ME, and 1 M NaCl, pH 5.0) over 120 min. Fractions with peptide cyclase activity were pooled and further subjected to size-exclusion chromatography with a HiLoad 16/600 Superdex 75 prep grade column at a flow rate of 0.8 ml/min. SDS-PAGE and Coomassie Blue staining were used to analyze enzyme purity.

### RNA isolation and transcriptome sequencing

RNA was isolated from fresh *M. cochinchinensis* seeds by a protocol based on that described by Djami-Tchatchou and Straker using CTAB extraction buffer (2% cetyltrimethylammonium bromide, 2% polyvinylpyrrolidone, 100 mM Tris-HCl (pH 8.0), 2 mM EDTA, 2 M NaCl, 2% β-ME). RNA library construction was performed using 1 μg of total RNA (RIN value >7.0) with an Illumina TruSeq mRNA Sample Prep kit (Illumina, Inc). Briefly, poly-A-containing mRNA molecules were purified using poly-T-attached magnetic beads. Following purification, mRNA fragmentation was performed using divalent cations at elevated temperatures. RNA fragments were reverse-transcribed into first-strand cDNA using SuperScript II reverse transcriptase (Invitrogen) and random primers, followed by second-strand cDNA synthesis using DNA Polymerase I and RNase H. The cDNA fragments were subjected to end repair processing, addition of a single “A” base, and ligation of the indexing adapters. The products were then purified and enriched using PCR to create the final cDNA library. The libraries were quantified using qPCR according to the qPCR Quantification Protocol Guide (KAPA Library Quantification kits for Illumina Sequencing platforms) and qualified using TapeStation D1000 ScreenTape (Agilent Technologies). Indexed libraries were sequenced at the Beijing Genomics Institute (China) using the HiSeq2500 platform (Illumina).

### Sequencing of native McPAL1 by LC-MS/MS

Isolated SDS-PAGE gel bands containing McPAL1 was reduced with 5 mM DTT and alkylated using 20 mM iodoacetamide at pH 7.0 at 37 °C. In-gel digestion of the intact proteins was performed with 10 μg/ml trypsin (Pierce, MS grade, Thermo Scientific) at pH 7.8 at 30 °C overnight or 15 μg/ml chymotrypsin (Pierce, MS grade, Thermo Scientific) at pH 7.8 at 37 °C overnight. Digested peptides were extracted from gel pieces with 50% acetonitrile (0.1% formic acid). Solvents were completely removed using Speedvac and peptides were redissolved in 0.1% formic acid in Milli-Q water. Preliminary sequencing was performed using MALDI-TOF-TOF (Applied Biosystems, 4800) and analyzed with MASCOT Daemon using the long open reading frames extracted from *M. cochinchinensis* seed transcriptome by TransDecoder version 5.1.1 (https://github.com/TransDecoder) as templates for database search where 50 ppm MS and 0.5 Da MS/MS tolerances were applied. A galactose-specific lectin was identified in the fraction with McPAL activity (Fig. S7). To obtain a better resolution, digested peptide samples prepared with the same method were subjected to LC-MS/MS sequencing on a UHPLC (Dionex UltiMate 3000, Thermo Scientific Inc) linked to a mass spectrometry (Orbitrap Elite, Thermo Scientific Inc). Fragmentation of peptides was by higher-energy CID. Resultant spectra from both trypsin and chymotrypsin digested samples were combined and analyzed using PEAKS studio (version 7.5, Bioinformatics Solutions) with 10 ppm MS and 0.05 Da MS/MS tolerances. Translated mRNA sequences encoding McPAL1-4 were used as templates for Database search. Peptide fragments for McPAL1 were summarized in Table S1.

### Recombinant expression, purification, and activation of McPAL1 from *E. coli*

DNA encoding full-length *M. cochinchinensis* McPAL1 without the putative signaling domain (residues Lys37-Ala490) was cloned and expressed as an ubiquitin fusion protein in T7 shuffle *E. coli* cells (New England BioLabs). Transformed cells were grown at 30 °C in LB broth to mid-log phase whereupon the temperature was lowered to 16 °C, and expression was induced with 1 mM isopropyl–D-1-thiogalactopyranoside (IPTG) for 20 h. Cells were harvested by centrifugation and resuspended in lysis buffer (50 mM Tris-HCl, 150 mM NaCl, 1 mM EDTA, 0.1% Triton-X100, 1 mM PMSF, pH 7.5), followed by sonication and removal of cellular debris by centrifugation.

Lysates containing recombinant McPAL1 were further purified by metal affinity using a Ni-NTA column (Bio-Rad). His tag-containing proteins bound to the column were eluted using 50 mM Tris-HCl, 300 mM imidazole, pH 8.0. Ni-NTA elution fractions containing the protein were dialyzed overnight and loaded onto 5 ml HiTrap Q Sepharose high-performance columns (GE Healthcare). Bound proteins were eluted with a continuous salt gradient of 0 to 30% buffer B (20 mM Bis-Tris, 2 M NaCl, pH 7) for 15 column volumes.

To self-activate McPAL1, 1 mM EDTA and 1 mM Tris(2-carboxyethyl)phosphine hydrochloride were added, and the pH was adjusted to 4.5 using glacial acetic acid before incubation for 5 h at 37 °C. The supernatant was purified using an SEC column (elution buffer containing 100 mM citric buffer, 50 mM NaCl, pH 4.5). Protein concentration was measured by UV absorbance at 280 nm.

### Recombinant expression of McPAL1 in Sf9-insect cells

A baculovirus expression vector system was used to produce the secreted form of McPAL1 in Sf9 cells. The cDNA sequence encoding McPAL1 residues Lys37 to Ala490 was cloned into a donor plasmid modified from the pDP1381 plasmid (46), which was a generous gift from Dominic Esposito. Briefly, the open reading frame encoded an N-terminal gp64 secretion signal, a hexahistidine tag, and tobacco etch virus protease site fused to the McPAL1 sequence. The L21 5′-UTR sequence (47), which can increase protein expression, was added between the late polyhedrin promoter and the open reading frame. Cloning and propagation of the donor plasmid were done in the *E. coli* DE14 strain (46) with ampicillin selection. We used the DE32 strain (48), which was also provided by Dominic Esposito, to produce the Δ(chitinase-cathepsin) baculoviral bacmid. Verified donor plasmid was transformed into chemically competent DE32 cells using a heat shock method. Cells recovered in SOC media for 2.5 to 3 h before they were used to directly inoculate 2 ml LB supplemented with kanamycin and incubated overnight at 37 °C with shaking. We bypassed plating and blue-white colony screening as this combination of donor plasmid and bacmid production strain routinely yields >95% white colonies. Recombinant bacmid was purified from the DE32 cell pellet using isopropanol precipitation and finally resuspended in 50 μl sterile water. After confirming the presence of the desired insert by PCR using polyhedrin promoter and M13 reverse primers, the bacmid was transfected into *Sf9*-cells using Cellfectin (Gibco) per the manufacturer's instructions. P0 baculovirus in the culture supernatant was collected 5 days posttransfection, supplemented with 10% fetal bovine serum (Gibco), and subsequently amplified to P2 virus that was used to infect *Sf9* cells on a liter scale.

McPAL1 purification was achieved using three chromatography steps. The media containing secreted zymogenic McPAL1 was centrifuged at 8000*g* for 20 min at 4 °C. The pH of the supernatant was then adjusted to 7.5 and injected into a GE Excel affinity purification column. After binding to the column, the protein was eluted using buffer A (20 mM HEPES pH 7.5, 150 mM NaCI, and 5 mM β-ME) and buffer B (20 mM HEPES pH 7.5, 150 mM NaCI, 5 mM β-ME, and 500 mM imidazole). The eluted target protein was then diluted tenfold using buffer A and further purified by ion exchange and size-exclusion chromatography.

Activation of zymogenic McPAL1 was performed at pH 4.5 for 16 h at 4 °C. N-lauroylsarcosine was added to the activation buffer to prevent religation of the cap domain to the core domain.

### Solid-phase synthesis of peptides

All peptides used in this study were synthesized on a Liberty Blue (CEM) automated microwave solid-phase synthesizer with Rink-amide resin (GL Biochem), using Fmoc/tBu chemistry. In the synthesis, five equivalents of the amino acid were used for each coupling cycle with PyBOP activation. A double-coupling protocol was performed if needed. All peptides were cleaved in 95% TFA/2.5% H2O/2.5% TIS cleavage solution and purified by prep-RP-HPLC.

### *In vitro* cyclization assays

Enzymatic reactions were performed in 30 to 40 μl reaction buffer (20 mM sodium phosphate/citrate, 1 mM DTT or TCEP, and 1 mM DTT) at 37 °C and a pH ranging from 4.0 to 8.0. After the reaction, the mixture was quenched using trifluoroacetic acid (TFA) to adjust the pH to <2. The quenched reaction mixtures were subjected to MALDI-TOF mass spectrometry using a C18 analytical column (Aries widespore). Peaks were collected, and the identity was characterized by either MALDI-TOF mass spectrometry or electrospray ionization (ESI).

### Reaction product analysis by MALDI-TOF MS

Molecular weights of all peptides characterized in this study were determined on an Applied Biosystem 5800 matrix-assisted laser desorption/ionization time-of-flight mass spectrometer (MALDI-TOF MS). The peptide samples were mixed 1:1 with CHCA (α-cyano-4-hydroxycinnamic acid) matrix prepared at 5 to 6 mg/ml in 80% (v/v) acetonitrile and 0.1% (v/v) formic acid. The peptide matrix mixture (∼1 μl) was spotted. The acquired spectra were analyzed using Data Explorer 4.1 software.

### Quantification of reaction products by RP-HPLC

Reaction mixtures were quantitatively analyzed using analytical-graded C18 column (Phenomenex, Aeris, 3.6 um, C-18, 200 Å, 250 × 21.2 mm) for reverse-phase HPLC (RP-HPLC). A general gradient used was 15 to 60% buffer B (acetonitrile with 0.1% TFA) from 3 to 20 min followed by 60 to 90% in 1 min.

## Data availability

Transcriptomic data for *M. cochinchinensis* seed extracts have been deposited with the National Center for Biotechnology Information (NCBI) under accession number PRJNA655570. The mass spectrometry proteomics data have been deposited to the ProteomeXchange Consortium *via* the PRIDE (http://www.ebi.ac.uk/pride) (49) partner repository with the dataset identifiers PXD028325 and PXD028327. All remaining data is presented in the main article and supporting information.

## Supporting information

This article contains supporting information (11, 49).

## Conflict of interest

The authors declare that they have no conflicts of interest with the contents of this article.
