# Supplementary material for: The legumain McPAL1 from Momordica cochinchinensis is a highly stable Asx-specific splicing enzyme
Source: J Biol Chem. 2021 Oct 26;297(6):101325. doi: 10.1016/j.jbc.2021.101325 (PMC8600085; doi:10.1016/j.jbc.2021.101325)
Supplement: Figures S1–S14 and Table S1 [file mmc1.docx]

­­

**SUPPORTING INFORMATION**

**The legumain McPAL1 from *Momordica cochinchinensis* is a highly stable Asx-specific splicing enzyme**


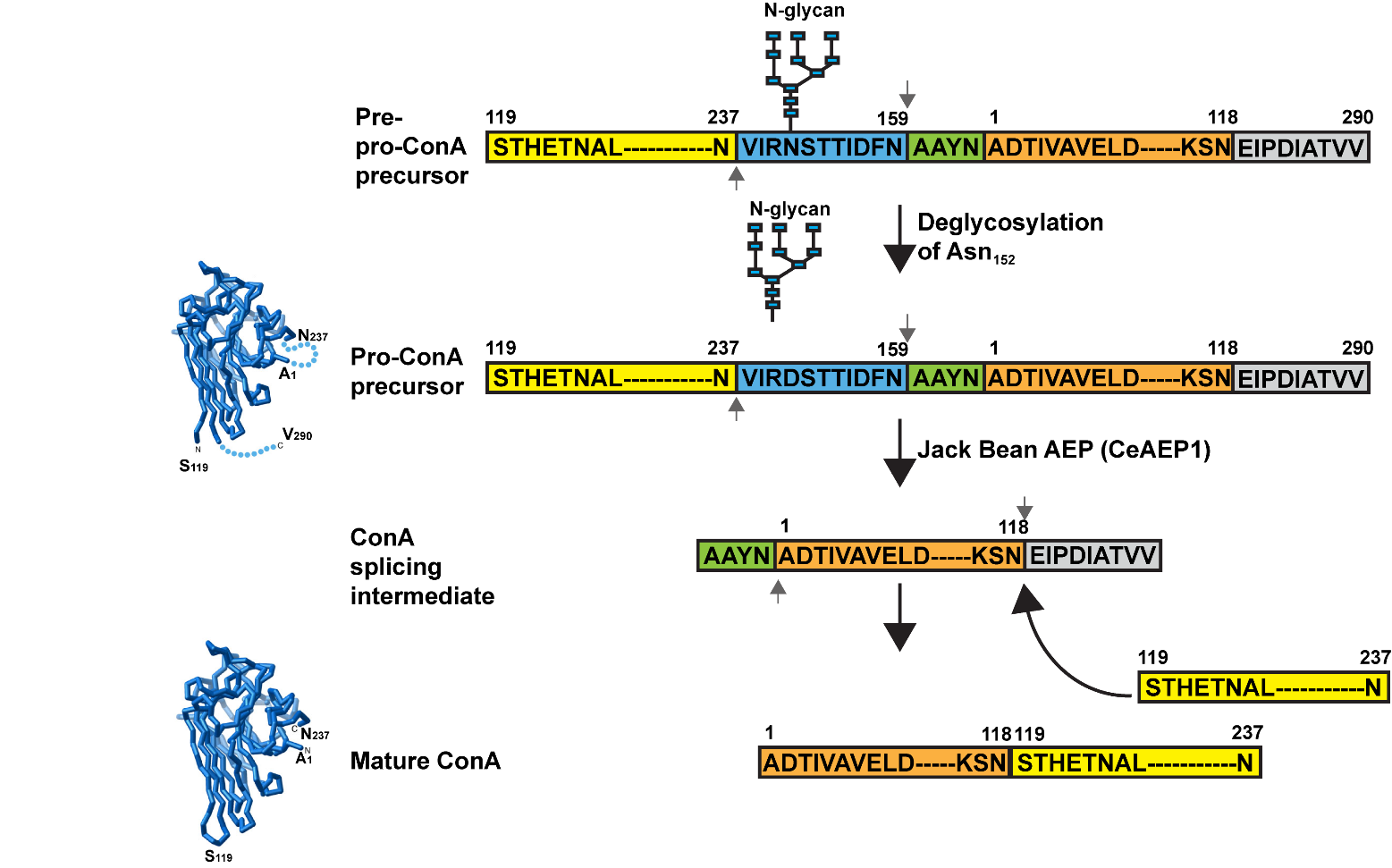


**Supplementary Figure S1. AEP-mediated Concanavalin A (ConA) splicing in *Canavalia ensiformis* (Jack Bean).** The gray arrows demonstrate the CeAEP1-cleavage sites. The incoming protein fragment (S_119_ to N_237_) (yellow) replaces the leaving fragment (gray) and ligates to N_118_, where the peptide bond forms between the N_118_ and S_119_ to generate the mature ConA. Numbering scheme on the precursor is based on the mature ConA according to pioneering work (1,2).


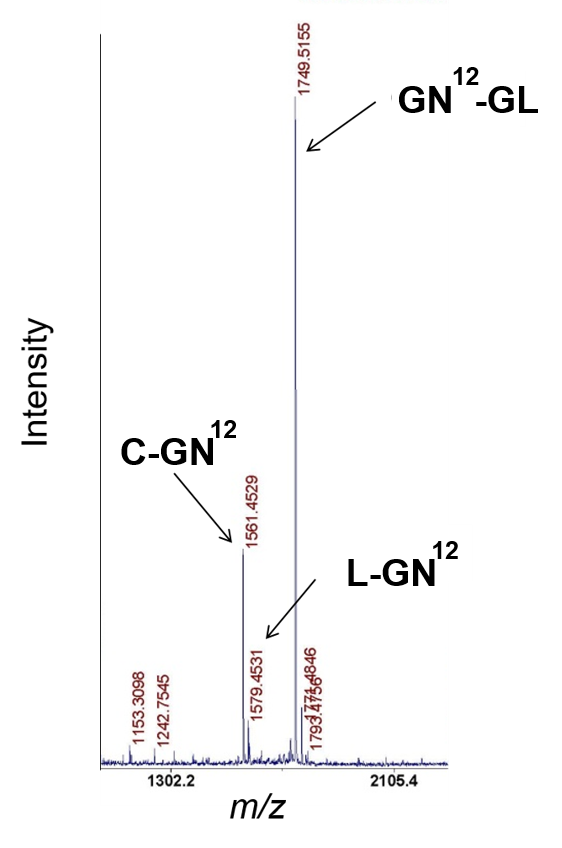


**Supplementary Figure S2.** **MALDI-MS profile of cyclization of peptide substrate GN^12^-GL by seed extract of *M. cochinchinensis.*** Substrate: GN^12^-GL, calc. MW=1749.0, obs. MW=1749.5. Cyclic product: c-GN^12^**,** calc. MW=1560.9, obs. MW=1561.4. Hydrolysis product: L-GN^12^: calc. MW=1578.9, obs. MW=1579.4.


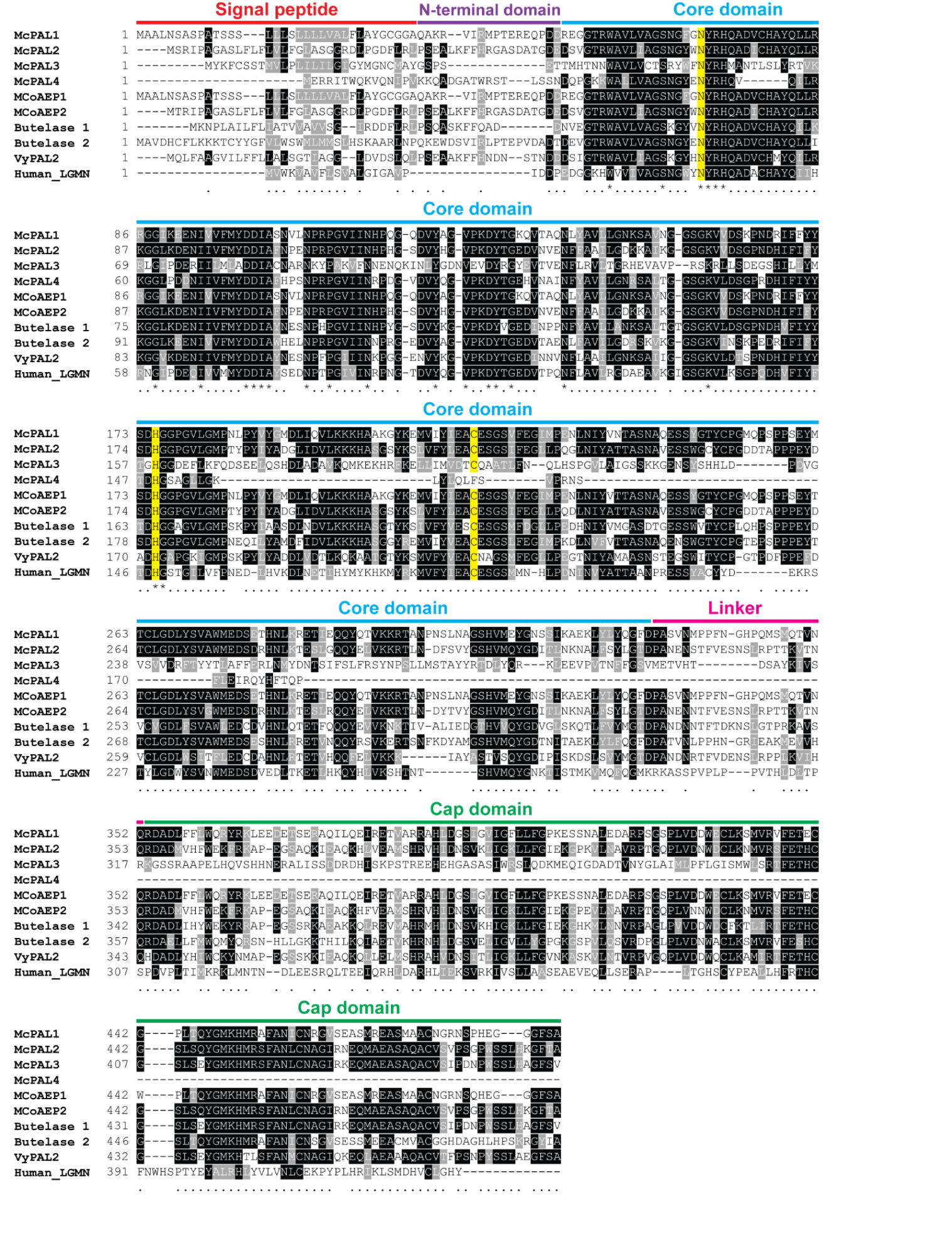


**Supplementary Figure S3.** **Multiple sequence alignment of the four putative McPAL sequences (McPAL1-4) discovered in the *M. cochinchinensis* transcriptome with legumain-like proteins butelase-1, VyPAL2 and human legumain (LGMN).** Putative protein domains are labelled. Black and gray boxes indicate conserved residues and conservative mutations, respectively. White indicates divergence. The conserved catalytic triad Asn70, His175 and Cys217 (numbering according to McPAL1) is shaded in yellow.


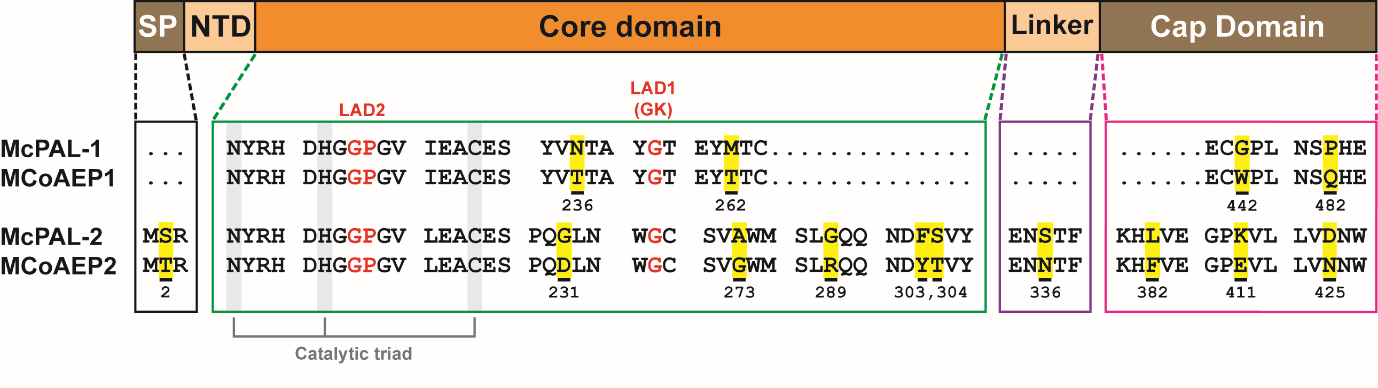


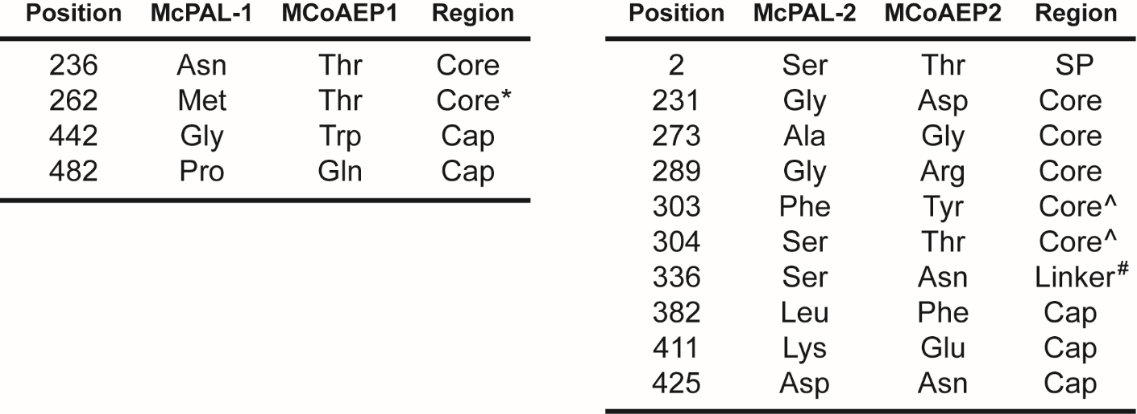


**Supplementary Figure S4. Sequence alignment of McPAL1 vs. MCoAEP1 and McPAL2 vs. MCoAEP2.** The predicted protein domains as indicated and LAD1/LAD2 regions are texted in red. Catalytic triad residues are shaded in gray. Differences between McPAL1 vs. MCoAEP1 and McPAL2 vs. MCoAEP2 are shaded in yellow.

**
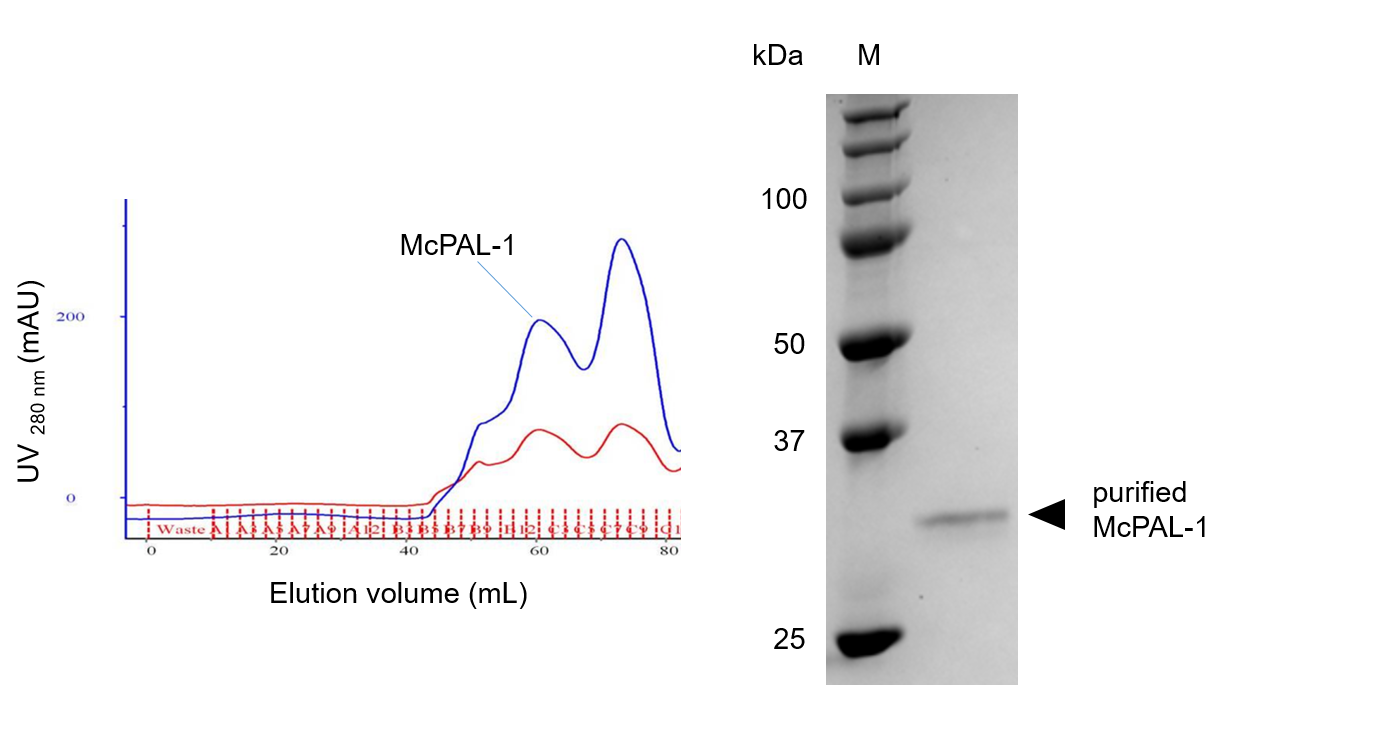
**

**Supplementary Figure S5.** **Size exclusion FPLC profile (left) of McPAL1 purification and SDS-PAGE of purified McPAL1 (right, reproduced from main Figure 1B for direct comparison with the FPLC profile)**.

**Supplementary Table 1. Peptide fragments of McPAL1 detected by LC-MS/MS in the seed extract of *M. cochinchinensis*.**

| **Peptide** | **Unique** | **-10lgP*** | **Mass** | **Length** | **ppm** | **m/z** | **z** | **RT** | **Area Sample** | **Scan** | **Source File** | **PTM** |
| --- | --- | --- | --- | --- | --- | --- | --- | --- | --- | --- | --- | --- |
| R.PGVIINHPQGQDVYAGVPK.D | Y | 120.59 | 1988.048 | 19 | 2.8 | 663.6918 | 3 | 20.15 | 2.23E+07 | 3019 | 180130_M1T.raw |  |
| R.WAVLVAGSNGFGNYR.H | Y | 113.96 | 1609.8 | 15 | -0.5 | 805.9069 | 2 | 32.85 | 8.30E+05 | 5109 | 180130_M1T.raw |  |
| R.WAVLVAGSN(+.98)GFGNYR.H | Y | 97.72 | 1610.784 | 15 | -0.3 | 806.399 | 2 | 35.73 | 1.71E+06 | 5590 | 180130_M1T.raw | Deamidation (NQ) |
| R.ETIEQQYQTVK.K | Y | 96.8 | 1365.678 | 11 | -0.7 | 683.8456 | 2 | 15.33 | 1.08E+07 | 2251 | 180130_M1T.raw |  |
| K.QVTAQNLYAVLLGNK.S | Y | 96.32 | 1630.904 | 15 | 1.9 | 816.4609 | 2 | 39.4 | 3.13E+05 | 6215 | 180130_M1T.raw |  |
| F.M(+15.99)YDDIASNVLNPRPGVIINHPQGQDVY.A | Y | 95.99 | 3040.471 | 27 | -2.5 | 1014.498 | 3 | 33.32 | 5.64E+05 | 5262 | 180130_M1C.raw | Oxidation (M) |
| F.MYDDIASNVLNPRPGVIINHPQGQDVY.A | Y | 95.61 | 3024.476 | 27 | -0.8 | 1009.168 | 3 | 34.69 | 8.84E+05 | 5460 | 180130_M1C.raw |  |
| K.Q(-17.03)VTAQNLYAVLLGNK.S | Y | 91.5 | 1613.878 | 15 | 2.2 | 807.9479 | 2 | 51.1 | 3.31E+04 | 8144 | 180130_M1T.raw | Pyro-glu from Q |
| Y.VYGMDLIQVLK.K | Y | 86.13 | 1277.705 | 11 | -1.6 | 639.8589 | 2 | 38.86 | 2.86E+05 | 6128 | 180130_M1T.raw |  |
| P.TEREQPDDREGGTRW.A | Y | 84.16 | 1830.825 | 15 | -2.1 | 611.2823 | 3 | 6.09 | 9.04E+05 | 904 | 180130_M1C.raw |  |
| L.VAGSNGFGNYR.H | Y | 83.69 | 1140.531 | 11 | 0.6 | 571.2733 | 2 | 12.45 | 5.50E+05 | 1812 | 180130_M1T.raw |  |
| R.HQADIC(+58.01)HAYQLLR.K | Y | 80.69 | 1624.778 | 13 | 6.6 | 542.6035 | 3 | 16.65 | 0 | 2479 | 180130_M1T.raw | Carboxymethyl |
| K.SAVN(+.98)GGSGK(+57.02)VVDSKPNDR.I | Y | 79.59 | 1843.902 | 18 | -0.4 | 461.9827 | 4 | 5.02 | 1.00E+05 | 609 | 180130_M1T.raw | Deamidation (NQ); Carbamidomethylation (DHKE X@N-term) |
| N.VLNPRPGVIINHPQGQDVY.A | Y | 75.99 | 2115.123 | 19 | -1 | 706.0491 | 3 | 21.23 | 2.94E+06 | 3280 | 180130_M1C.raw |  |
| Y.GMDLIQVLK.K | Y | 70.34 | 1015.574 | 9 | -1.4 | 508.7934 | 2 | 31.97 | 2.52E+05 | 4984 | 180130_M1T.raw |  |
| Y.VNTASNAQESSY.G | Y | 68.91 | 1269.547 | 12 | -2.4 | 635.781 | 2 | 9.82 | 3.27E+05 | 1479 | 180130_M1C.raw |  |
| F.YYSDHGGPGVLGMPNLPY.V | Y | 66.37 | 1935.882 | 18 | -3.9 | 968.9471 | 2 | 38.89 | 4.91E+04 | 6147 | 180130_M1C.raw |  |
| Y.YSDHGGPGVLGMPNLPY.V | Y | 65.9 | 1772.819 | 17 | -2.4 | 887.4169 | 2 | 37.87 | 3.46E+04 | 5995 | 180130_M1C.raw |  |
| Y.TGKQVTAQNLY.A | Y | 65.17 | 1221.635 | 11 | -1.6 | 611.8255 | 2 | 13.15 | 5.79E+06 | 2004 | 180130_M1C.raw |  |
| Y.SVAWM(+15.99)EDSETH.N | Y | 61.26 | 1306.514 | 11 | -1.2 | 654.2649 | 2 | 14.26 | 7.91E+05 | 2172 | 180130_M1C.raw | Oxidation (M) |
| L.RRGGIKEENIVVF.M | Y | 61.1 | 1515.852 | 13 | -2.1 | 758.9336 | 2 | 16.97 | 2.07E+06 | 2621 | 180130_M1C.raw |  |
| H.NLKRETIEQQY.Q | Y | 58.23 | 1420.731 | 11 | -4.8 | 711.3711 | 2 | 10.24 | 2.42E+06 | 1532 | 180130_M1C.raw |  |
| Y.SVAWMEDSETH.N | Y | 57.86 | 1290.519 | 11 | -1.5 | 646.2672 | 2 | 20.79 | 6.54E+05 | 3208 | 180130_M1C.raw |  |
| F.YYSDHGGPGVL.G | Y | 57.79 | 1163.525 | 11 | 1.2 | 582.7717 | 2 | 18.81 | 1.14E+05 | 2926 | 180130_M1C.raw |  |
| Y.VNTASNAQE(+21.97)SSYGTY.C | Y | 56.29 | 1612.649 | 15 | 1.9 | 807.3354 | 2 | 17.34 | 5.10E+04 | 2678 | 180130_M1C.raw | Replacement of 2 protons by magnesium |
| L.NPRPGVIINHPQGQDVY.A | Y | 55.98 | 1902.97 | 17 | -2.7 | 635.3304 | 3 | 15.77 | 9.07E+05 | 2582 | 180130_M1C.raw |  |
| F.EGIM(+15.99)PENLNIY.V | Y | 55.47 | 1307.607 | 11 | -1.1 | 654.8115 | 2 | 34.63 | 2.32E+05 | 5454 | 180130_M1C.raw | Oxidation (M) |
| Y.Q(-17.03)LLRRGGIKEENIVVF.M | Y | 55.01 | 1853.052 | 16 | -1.4 | 618.692 | 3 | 31.34 | 2.07E+05 | 4937 | 180130_M1C.raw | Pyro-glu from Q |
| R.ETIE(+21.98)QQYQTVK.K | Y | 53.51 | 1387.66 | 11 | -4.1 | 694.8342 | 2 | 15.33 | 4.97E+04 | 2270 | 180130_M1T.raw | Sodium adduct |
| T.EREQPDDREGGTRW.A | Y | 52.98 | 1729.777 | 14 | -0.8 | 577.6005 | 3 | 6.02 | 8.24E+04 | 833 | 180130_M1C.raw |  |
| Y.SVAWM(+15.99)EDSETHNLKR.E | Y | 50.11 | 1817.837 | 15 | 3.5 | 455.468 | 4 | 11.47 | 0 | 1662 | 180130_M1T.raw | Oxidation (M) |
| M.PTEREQPDDREGGTRW.A | Y | 48.84 | 1927.877 | 16 | -2.1 | 482.9767 | 4 | 7.49 | 2.57E+04 | 1100 | 180130_M1C.raw |  |
| F.YYS(-15.99)DHGGPGVLGMPNLPY.V | Y | 45.41 | 1919.888 | 18 | 0 | 960.9534 | 2 | 38.75 | 4.14E+04 | 6116 | 180130_M1C.raw | Deoxy |
| L.VAGSNGFGNY.R | Y | 45.37 | 984.4301 | 10 | -1.9 | 493.2226 | 2 | 20.26 | 1.57E+05 | 3153 | 180130_M1C.raw |  |
| L.RRGGIKEENIVV.F | Y | 44.28 | 1368.784 | 12 | -3.5 | 457.268 | 3 | 8.73 | 1.33E+05 | 1300 | 180130_M1C.raw |  |
| Y.VYGM(+15.99)DLIQVL.K | Y | 44.28 | 1165.605 | 10 | -3.2 | 583.8094 | 2 | 42.56 | 1.77E+05 | 6741 | 180130_M1C.raw | Oxidation (M) |
| P.T(+57.02)EREQPDDREGGTRW.A | Y | 42.63 | 1887.846 | 15 | -1.4 | 472.9693 | 4 | 6.58 | 1.63E+04 | 973 | 180130_M1C.raw | Carbamidomethylation (DHKE X@N-term) |
| R.HQADVC(+58.01)HAYQLLR(-.98).R | Y | 39.8 | 1609.778 | 13 | -2.4 | 805.8945 | 2 | 16.36 | 1.94E+05 | 2434 | 180130_M1T.raw | Carboxymethyl; Amidation |
| R.GGIKEENIVVFMYDDIASNVLNPR.P | Y | 38.54 | 2692.353 | 24 | 1.9 | 898.46 | 3 | 50.43 | 3.63E+05 | 8026 | 180130_M1T.raw |  |
| Y.TGKQVTAQNLY(+21.98).A | Y | 37.86 | 1243.617 | 11 | -3.4 | 622.8154 | 2 | 13.21 | 1.50E+05 | 2012 | 180130_M1C.raw | Sodium adduct |
| R.RGGIKEENIVVFMYDDIASNVLNPR.P | Y | 35.41 | 2848.454 | 25 | 0.3 | 713.121 | 4 | 45.21 | 4.67E+05 | 7174 | 180130_M1T.raw |  |
| K.LLFGIEK.G | Y | 34.65 | 818.4902 | 7 | 3.2 | 410.2537 | 2 | 25.9 | 4.59E+05 | 3937 | 180130_M1T.raw |  |
| K.SAVNGGSGK(+41.03)VVDSKPNDR.I | Y | 34.65 | 1826.924 | 18 | 1.1 | 457.7386 | 4 | 4.89 | 2.05E+04 | 605 | 180130_M1T.raw | Amidination of lysines or N-terminal amines with methyl acetimidate |
| L.KKKHAAKGYKEM(+15.99)VIY.I | Y | 34 | 1808.997 | 15 | -2.4 | 453.2566 | 4 | 1.64 | 1.28E+04 | 200 | 180130_M1C.raw | Oxidation (M) |
| L.VAGSN(+.98)GFGNYR.H | Y | 28.25 | 1141.515 | 11 | 0.7 | 571.7653 | 2 | 14.4 | 1.71E+05 | 2103 | 180130_M1T.raw | Deamidation (NQ) |
| Y.TGKQ(+.98)VTAQNLY.A | Y | 26.07 | 1222.619 | 11 | 2 | 612.3197 | 2 | 34.76 | 6.48E+05 | 5491 | 180130_M1C.raw | Deamidation (NQ) |
| R.VIRMPT(+114.04)EREQPDDREGGTRW.A | Y | 26.01 | 2541.214 | 20 | 10 | 636.3187 | 4 | 3.39 | 2.39E+05 | 483 | 180130_M1C.raw | Ubiquitin |
| R.VIRMPTEREQ(+.98)PDDREGGTRW.A | Y | 25.29 | 2428.155 | 20 | 0.2 | 608.0477 | 4 | 1.77 | 1.29E+05 | 216 | 180130_M1C.raw | Deamidation (NQ) |

*FDR cut-off: -10lgP > 25.

**
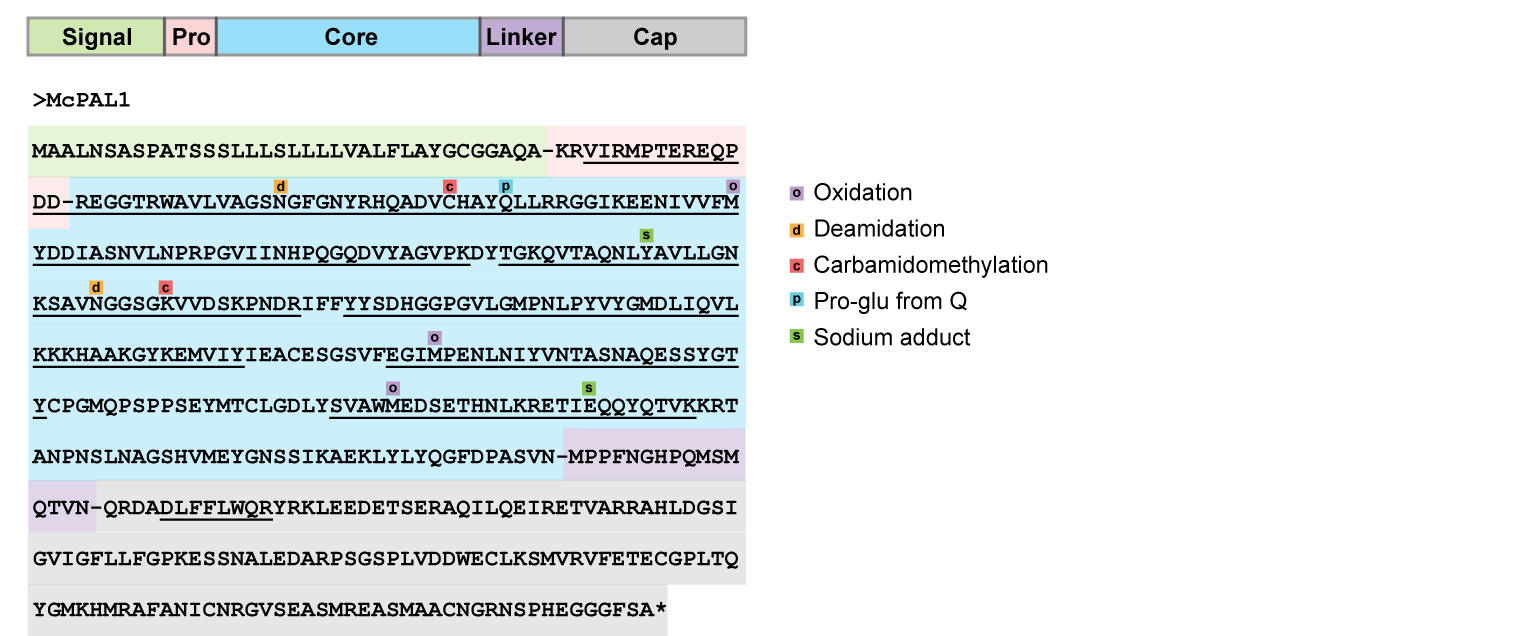
**

**Supplementary Figure S6. Translated sequence of McPAL1 from *M. cochinchinensis* and regions identified by LC-MS/MS.** The sequence is color-coded, with the endoplasmic reticulum signal shown in green, the N-terminal prodomain in red, the AEP core domain in blue, the linker in purple, and the cap domain in grey. Peptide sequences identified from in-gel digestion and MS/MS sequencing are underlined. Core-domain sequence coverage was 73.6%. Data have been deposited to the ProteomeXchange Consortium via the PRIDE (3) partner repository with the dataset identifier PXD028327.


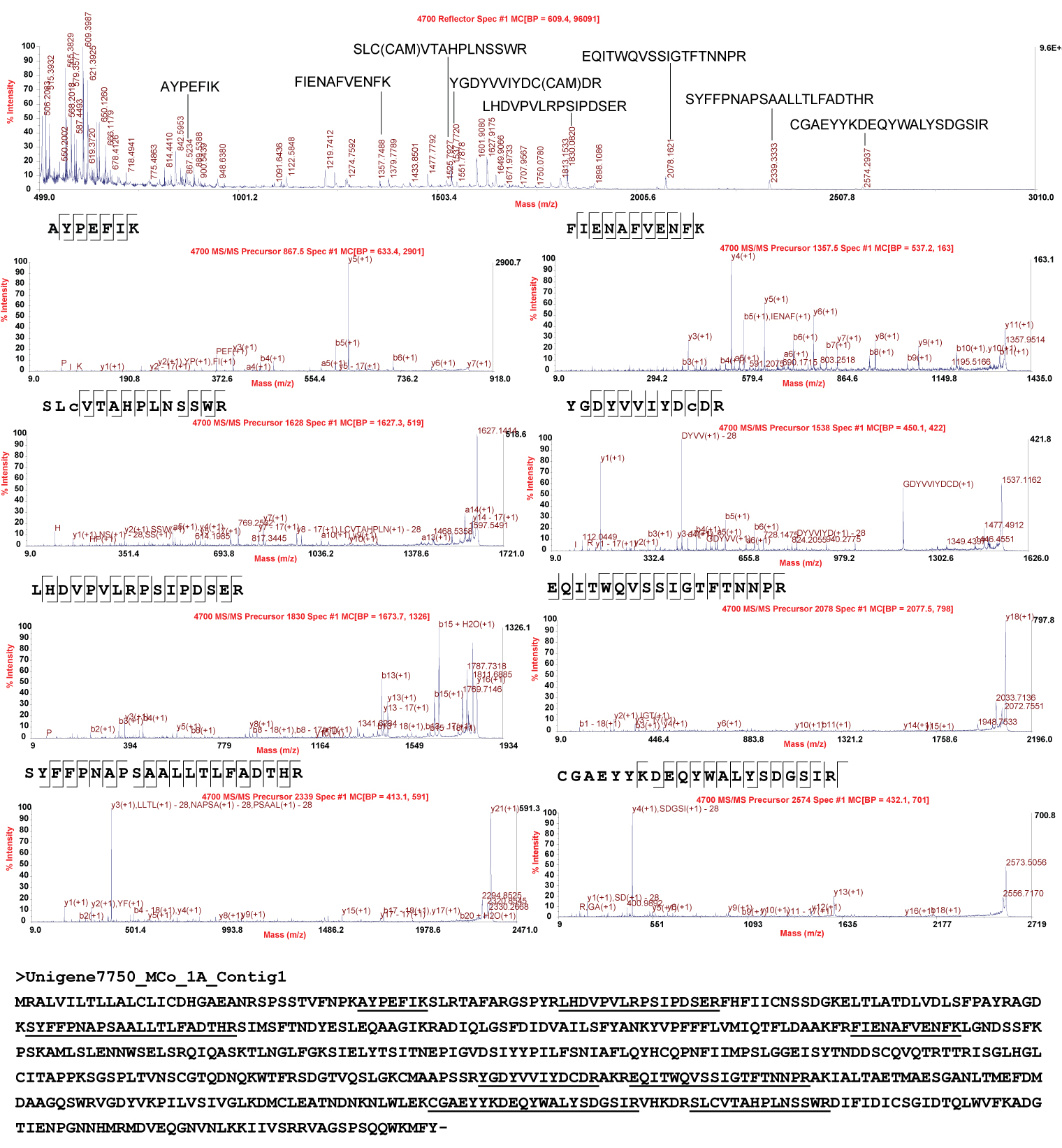


**Supplementary Figure S7.** MS/MS sequencing result of the McPAL1-containing sample by MALDI-TOF-TOF. A few tryptic-digested peptide fragments were identified using transcriptome database and matched with a seed lectin. Data have been deposited to the ProteomeXchange Consortium via the PRIDE (3) partner repository with the dataset identifier PXD028325.


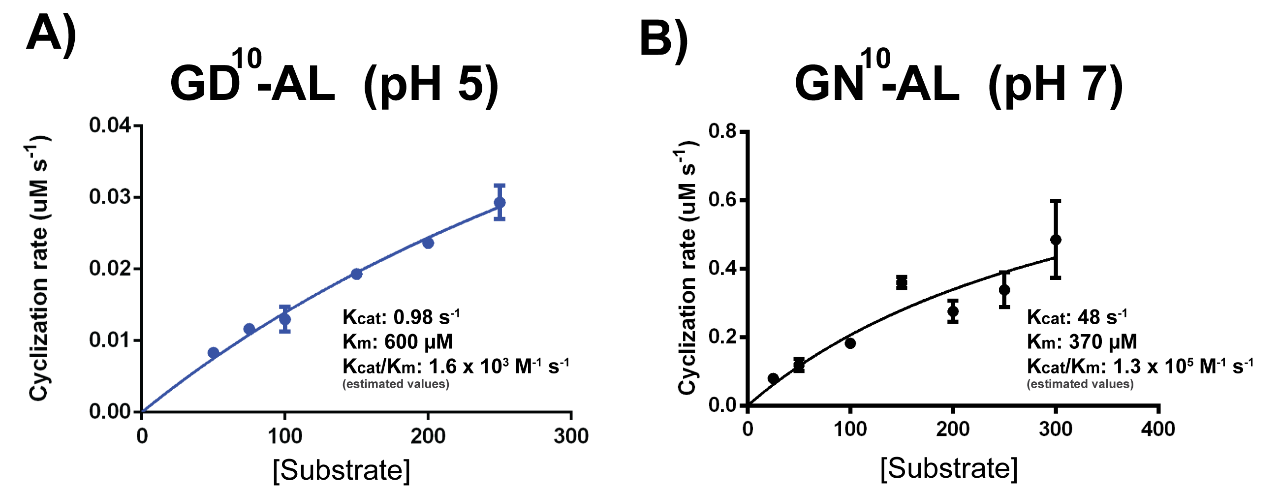


**Supplementary Figure S8. Michaelis-Menten plots of kinetics for the cyclization of (A) GD^10^-AL and (B) GN^10^-AL by McPAL1.** 50 uM of McPAL1 was incubated with substrates of different concentrations at pH 5 for **GD^10^-AL** and pH 7 for **GN^10^-AL**. Kinetic parameters are estimated values.

**
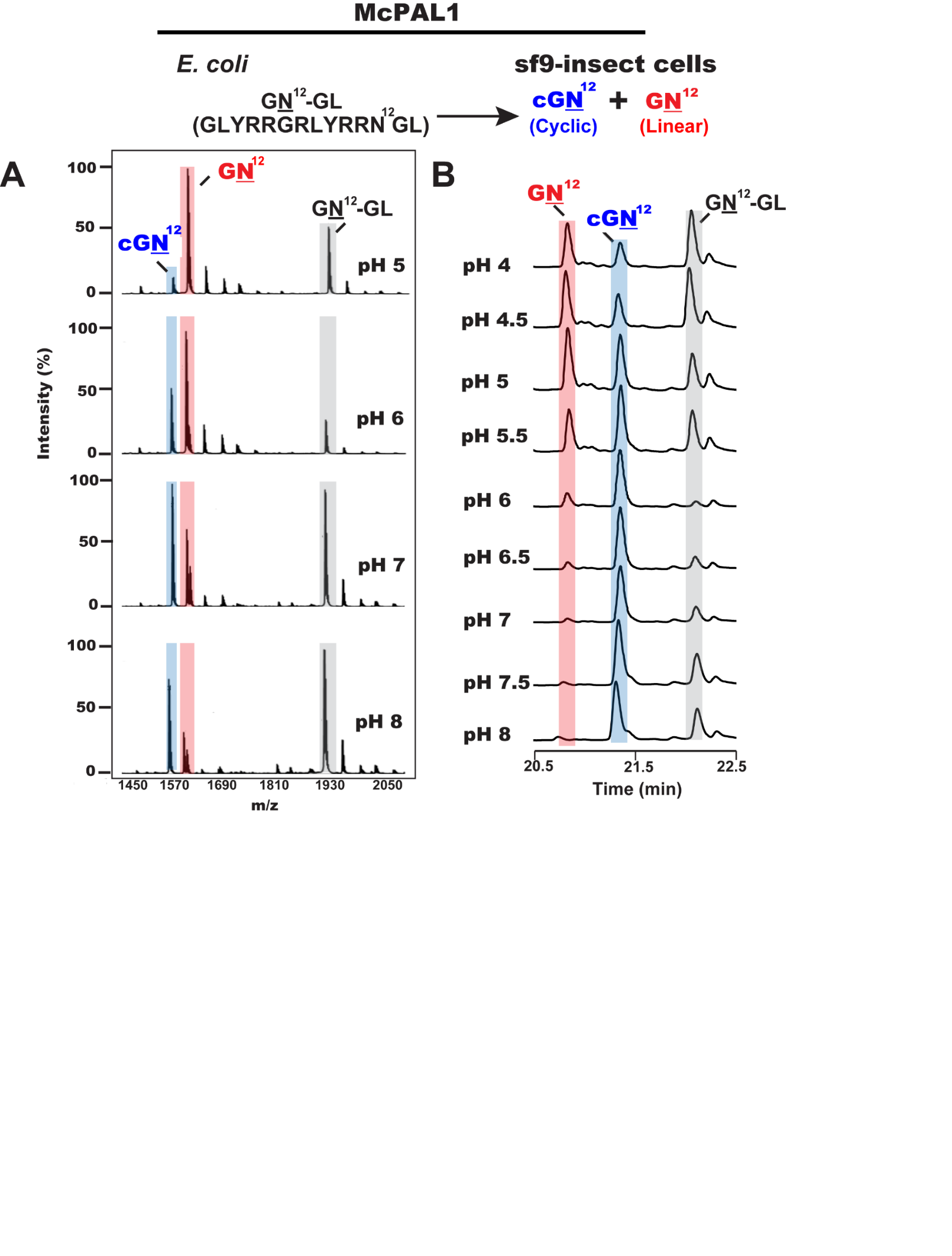
**

**Supplementary Figure S9. Product analysis of hydrolysis and cyclization of peptide substrate P1-Asn-containing peptide GN^12^-GL from pH 4-8 by McPAL1 expressed from *E.coli* or *Sf9* insect cells.** MALDI-TOF mass spectrometry and HPLC profiles of peptide cyclization and hydrolysis mediated by McPAL1 produced from **(A)** *E. coli* or **(B)** *Sf9* insect cells. Cyclic and linear GN^12^ are shaded in blue and pink, respectively. The starting material GN^12^-GL is shaded in gray. HPLC gradient: 15-60%,10-30 min.


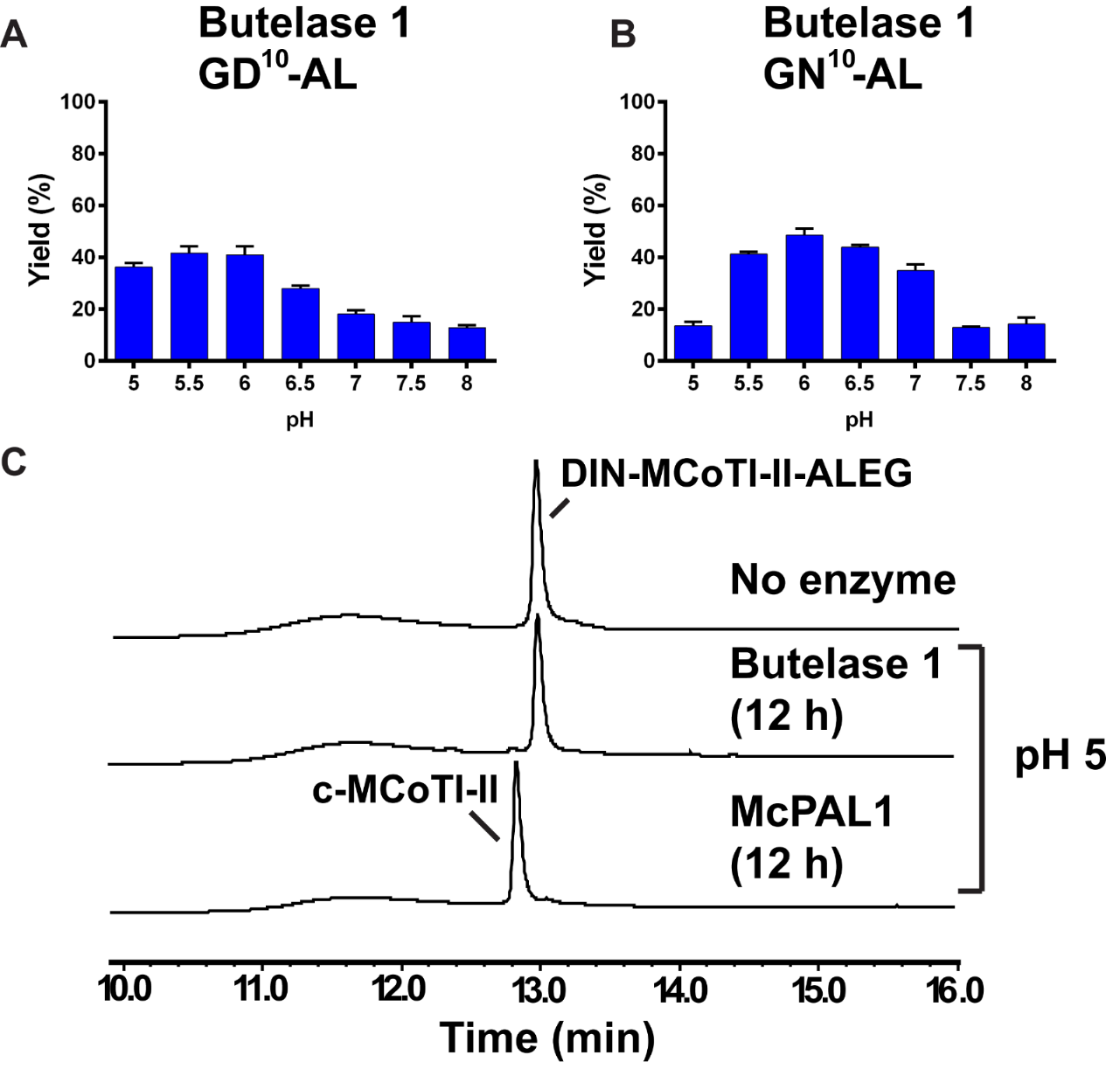


**Supplementary Figure S10. Characterization of butelase-1 on the P1-Asp, P1-Asn-containing substrates, and MCoTI-II precursor. (A, B)** Butelase-1 was incubated with peptide substrates at pH 5-8 with an enzyme-to-substrate ratio of 1:1500 and 1:200 for **GN^10^-AL** and **GD^10^-AL**, respectively. **(c)** Butelase-1 and McPAL1 were incubated with DIN-MCoTI-II-ALEG for 12 hr at pH 5, with and enzyme-to-substrate ratio of 1:200. Average yield and SDs were calculated from experiments performed in triplicates. HPLC gradient: 15-60%, 3-20 min.

**
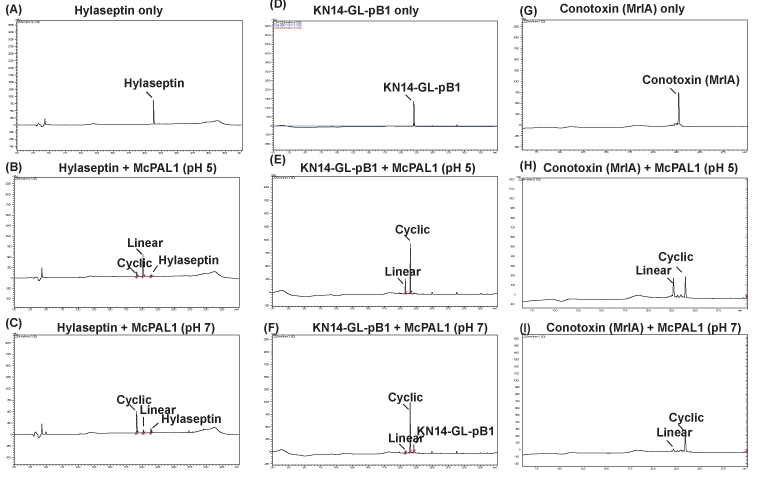
**

**Supplementary Figure S11. Raw HPLC-profiles of McPAL1-mediated cyclization of hylaseptin, KN^14^-GL-pB1 and conotoxin (MrlA).** The reactions were performed at pH 5 and 7 for 10 min with an enzyme-to-substrate ratio of 1:1500 at 37°C. HPLC gradient: 15-60%,10-30 min.


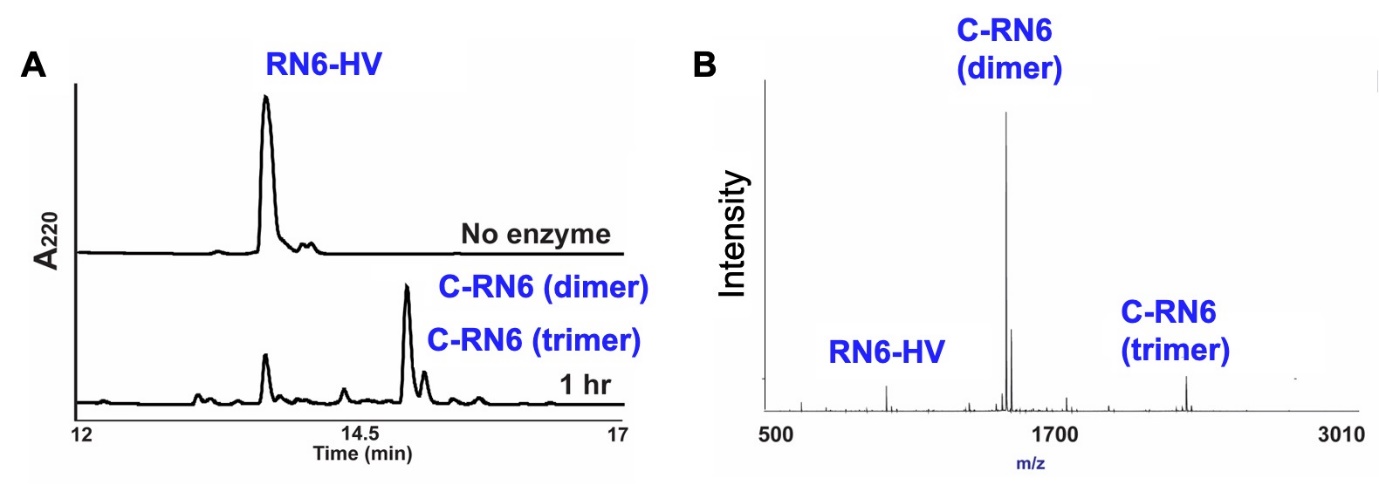


**Supplementary Figure S12. McPAL1-mediated cyclo-oligomerization of P1-Asn-containing peptides. (A)** HPLC profile and **(B)** mass spectrometry analysis of cyclo-oligomerization of RLYR-containing motif RN^6^-HV (MW. 1013.6 Da) at pH 7 to produce cyclodimer (MW. 1518.8 Da) and cyclotrimer (MW. 2278.2 Da). HPLC gradient: 15-60%, 3-20 min.

**
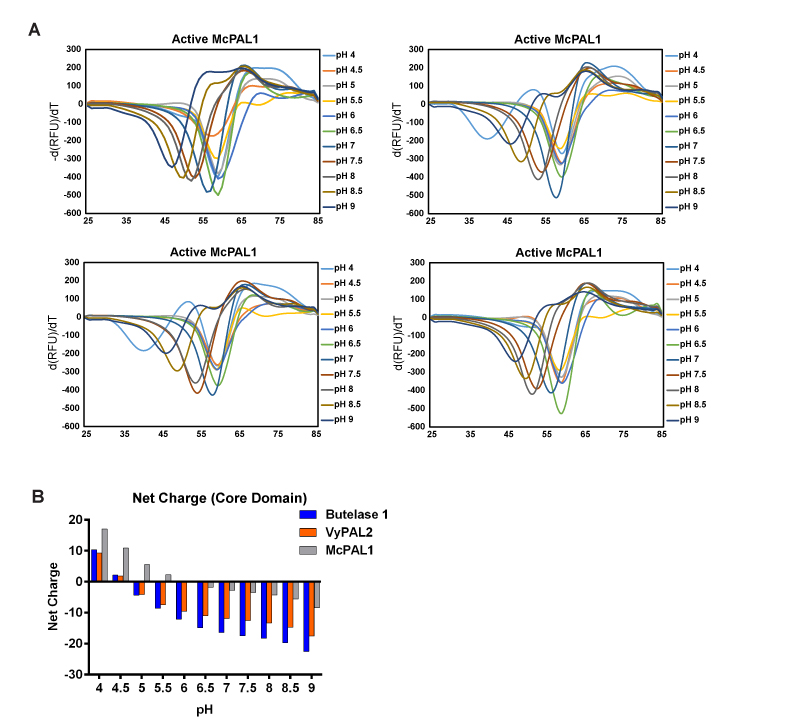
**

**Supplementary Figure S13. Thermal stability and net charge of McPAL1 at pH 4 to 9.** **(A)** Thermal stability assay of active McPAL1 at pH 4-9 with four replicates. **(B)** Net charge of active McPAL1, butelase-1, and VyPAL2, at pH 4-9.


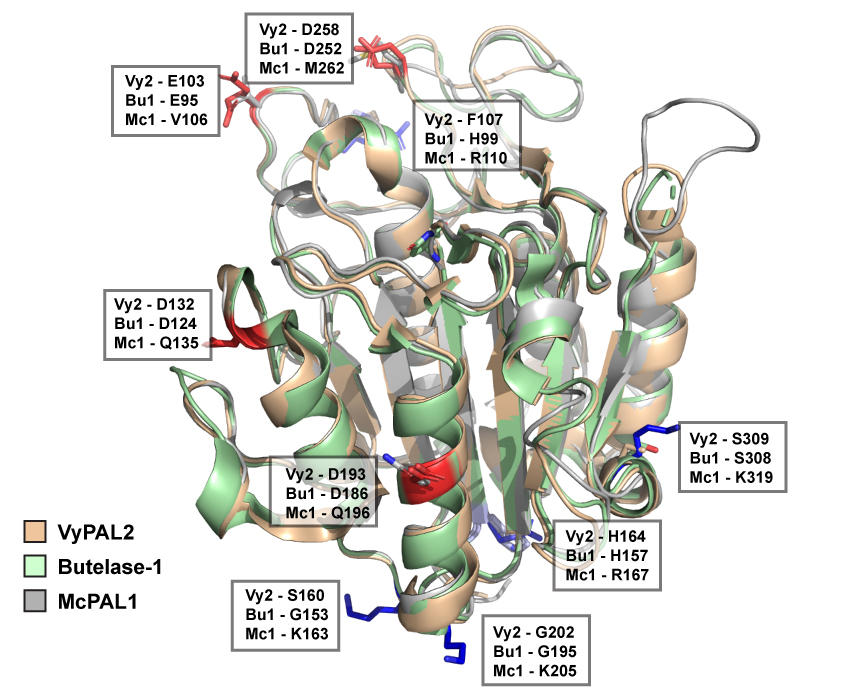


**Supplementary Figure S14. Difference in surface charges among McPAL1 (Mc1), butelase-1 (Bu1) and VyPAL2 (Vy2).** Locations of charged residues on McPAL1 in contrast to VyPAL2 and butelase-1.

**References**

1. Bowles, D. J., Marcus, S. E., Pappin, D. J., Findlay, J. B., Eliopoulos, E., Maycox, P. R., and Burgess, J. (1986) Posttranslational processing of concanavalin A precursors in jackbean cotyledons. *The Journal of cell biology* **102**, 1284-1297

2. Min, W., and Jones, D. H. (1994) In vitro splicing of concanavalin A is catalyzed by asparaginyl endopeptidase. *Nature Structural Biology* **1**, 502-504

3. Perez-Riverol, Y., Csordas, A., Bai, J., Bernal-Llinares, M., Hewapathirana, S., Kundu, D. J., Inuganti, A., Griss, J., Mayer, G., Eisenacher, M., Pérez, E., Uszkoreit, J., Pfeuffer, J., Sachsenberg, T., Yilmaz, Ş., Tiwary, S., Cox, J., Audain, E., Walzer, M., Jarnuczak, A. F., Ternent, T., Brazma, A., and Vizcaíno, J. A. (2019) The PRIDE database and related tools and resources in 2019: Improving support for quantification data. *Nucleic Acids Research* **47**, D442-D450
